# Supplementary material for: Online stroke forum as source of data for qualitative research: insights from a comparison with patients’ interviews
Source: BMJ Open. 2018 Mar 30;8(3):e020133. doi: 10.1136/bmjopen-2017-020133 (PMC5884329; doi:10.1136/bmjopen-2017-020133)
Supplement: Author's manuscript [file bmjopen-2017-020133.draft_revisions.pdf]

BMJ Open is committed to open peer review. As part of this commitment we make the peer review history of every article we publish publicly available.

When an article is published we post the peer reviewers' comments and the authors' responses online. We also post the versions of the paper that were used during peer review. These are the versions that the peer review comments apply to.

The versions of the paper that follow are the versions that were submitted during the peer review process. They are not the versions of record or the final published versions. They should not be cited or distributed as the published version of this manuscript.

BMJ Open is an open access journal and the full, final, typeset and author-corrected version of record of the manuscript is available on our site with no access controls, subscription charges or pay-per-view fees (http://bmjopen.bmj.com).

If you have any questions on BMJ Open's open peer review process please email info.bmjopen@bmj.com

BMJ Open

An online stroke forum as source of data for qualitative research: insights from a comparison with patients' interviews

Journal:	*BMJ Open*
Manuscript ID	bmjopen-2017-020133
Article Type:	Research
Date Submitted by the Author:	17-Oct-2017
Complete List of Authors:	JAMISON, JAMES; UNIVERSITY OF CAMBRIDGE, PUBLIC HEALTH & PRIMARY CARE<br>Sutton, Stephen; University of Cambridge,<br>Mant, Jonathan; University of Cambridge, General Practice and Primary Care Research Unit<br>De Simoni, Anna; Queen Mary University of London, Centre for Primary Care and Public Health
**Primary Subject Heading**:	Research methods
Secondary Subject Heading:	Qualitative research, Research methods, Cardiovascular medicine
Keywords:	STROKE MEDICINE, QUALITATIVE RESEARCH, Online forum, Methodology

SCHOLARONE™ Manuscripts

An online stroke forum as source of data for qualitative research: insights from a comparison with patients' interviews

*James Jamison[1]

Stephen Sutton[1]

Jonathan Mant[1]

Anna De Simoni[2]

[1]Primary Care Unit, Institute of Public Health, Forvie Site, University of Cambridge School of Clinical Medicine, Box 113 Cambridge Biomedical Campus, Cambridge, CB2 0SR.

[2]Centre for Primary Care and Public Health, Barts and the London School of Medicine and Dentistry, Queen Mary University of London, London, E1 2AB.

Email: a.desimoni@qmul.ac.uk Phone: Tel +44 (0)207 882 2520

*corresponding author: James Jamison; Tel: +44 (0)1223 768272. Fax: +44 (0)1223 763492. Email: jj285@medschl.cam.ac.uk

For peer review only

Abstract

Objective

To compare barriers and facilitators to adherence to secondary prevention medications after stroke using two data sources, qualitative interviews and online forum posts; to determine the appropriateness of an online forum as a source of data for qualitative research on medicine taking after stroke.

Design

Comparison of two independent studies, one using semi-structured interviews and the other an online forum, as a method of data collection. Thematic analysis in both studies informed by the Perceptions and Practicalities Approach.

Setting

Interviews were conducted in UK GP practices in 2013 and 2014; Online posts were written by UK stroke survivors and family members taking part in the online forum of the Stroke Association between 2004 and 2011.

Participants

42 interview participants (28 stroke survivors, 14 caregivers) and 84 participants to the online forum (49 stroke survivors, 33 caregivers, 2 unknown).

Results

Participants' characteristics of forum users were often missing. The median age of interview survivors was 72 years and 50 years for forum survivors. Most forum participants had experienced a stroke within the previous 12 months, while interviewees had done so one to five years previously.

All the key themes that emerged from the interviews could be matched with corresponding themes from the forum. Compared with interviews, the forum yielded additional themes such as the influence of bad press on statins on taking medications, criticisms of clinicians' prescribing practices, and the

For peer review only

burden of caregivers in assisting patients with medications and in being advocates for stroke survivors with healthcare professionals.

Conclusions

This investigation shows an online stroke forum is an appropriate source of data for qualitative research on patients' and caregivers' issues with adherence to secondary prevention medications after stroke. An online forum may offer additional insights compared to interviews, which can be attributed to differences in the approach to data collection.

Abstract word count- 300

Keywords: Stroke, Online forum, Qualitative interviews, Methodology

For peer review only

Article summary

Strengths and Limitations

- To our knowledge this is the first attempt to compare two different data sources to identify barriers to adherence to secondary prevention medications after stroke - a UK online forum and semi-structured interviews.
- All the key themes that emerged from the interviews could be matched with corresponding themes from the online forum, reassuring researchers that an online stroke forum is a valid source of data in its own right.
- Understanding of themes was not affected by potential limitations of an online forum such as lack of knowledge of participant characteristics, absence of face to face interaction and inability to directly question participants.
- A limitation of this study is the inability to compare the two sources in respect of participants' characteristics such as type of stroke and geographical location.
- With no control over the direction of discussions, the effectiveness of the online forum as a source of data will depend on how well the forum posts address the research question.

Funding: Anna De Simoni is funded by a NIHR Academic Clinical Lectureship. James Jamison was supported by a research grant from The Stroke Association and the British Heart Foundation: TSA BHF 2011/01

Data sharing statement: No additional data available.

For peer review only

Introduction

In recent years participation in online forums has increased dramatically out of a need for patients to know more about the health care conditions they face. [1 2 3 4 5] With the use of online health forums steadily increasing, greater efforts are being made to understand this mode of data collection for qualitative research. [6 7] Online patients' communities represent an important source of information offering access to hard-to-reach groups who are often excluded (or exclude themselves) from traditional research studies. [8 9]

Internet use across the UK has grown considerably. A recent report on Internet usage found that in the first quarter of 2017, approximately 89% of adults used the Internet, increasing from 52% in 2011 to 78% in 2016 for those between the ages of 65 and 74.[10] 42% of those 75 years and older of all genders are now Internet users.[11] It is estimated that one in four people with a chronic condition who use the Internet go online to find others with similar health concerns.[12] Patients engage with the Internet to access health information [13] and manage chronic illness.[14]

Patients are becoming more informed about their health through using the Internet.[15 16] Around 70% of Europeans who access the Internet, using it to obtain health information.[17] In the UK, digital technology has recently emerged as a key vehicle for the delivery of health and social care. [18] A review of the use of technology in healthcare confirmed that social media were increasingly used to communicate health information among public, patients and health professionals. [19]

As a method of capturing data on health attitudes and behaviour, the online forum offers considerable advantages, including access to large numbers of prospective participants with the potential for open and honest discussions.[20 8 9 21] Such forums have been used previously in health care research [22] across a range of health domains. [23 24 25 26]

In the face of increased technological change, there is a growing need to understand the potential for online sources of qualitative data [27] and their advantages and disadvantages compared with traditional data collection techniques.[28 29]

The face to face interview is an important qualitative data collection technique widely used in health care research. [30 31] The method permits close observation of respondents, flexibility to adapt the direction of conversation and the scrutiny of physical cues such as body language.

There is little research to date exploring the potential for online forums as a source of data collection compared with traditional qualitative techniques. Difficulties verifying participants' identity and medical condition (i.e. are forum users real patients with stroke?) as well as the inability to interpret visual cues and seek clarification to questions suggest that it may be necessary to confirm results with more established data sources, such as qualitative interviews. Confirmation of data may be deemed necessary in order to explore whether forum findings are representative of issues experienced by patients with stroke, and whether differences between the two sources could be used to decide which is better suited to addressing a particular research question. In an investigation comparing an online forum with qualitative interviews among cancer patients, the authors concluded that the forum offered useful data for qualitative health research.[32] Similarly, comparison between an online forum and face to face focus groups in people with multiple sclerosis concluded that forum results were comparable. [33] Comparison of characteristics of online versus face to face approaches have been reported more frequently with respect to focus groups.[34 35]

Drawing on a realistic evaluation perspective.[36] the present study explores differences between two approaches to data collection, interviews and an online stroke forum, by seeking to understand the attributes that underpin each data source and explore the context within which data collection occur.

For peer review only

Methods

Design

Comparison of themes around barriers and facilitators of adherence to secondary prevention medications after stroke in two independent studies, qualitative semi-structured interviews and an online forum. As interviews are a widely accepted method in qualitative research, [37] we used this as the standard against which to compare forum data. Differences and similarities in the data were examined, and results were compared and contrasted to explore the potential of the online forum as a data collection source.

Interviews-dataset

Interview participants included stroke survivors recruited through five General practice surgeries in Eastern England, along with their caregivers, as described previously [38]. To allow us to directly compare the analyses from these interviews and an online stroke forum [39], interview transcripts were re-analysed using a thematic analysis [40] according to the PAPA framework (see details in the section Procedure and Analysis).[41 42]

Using a thematic analysis approach permitted an in-depth exploration of the data following an essential/realist approach which took into account the experiences and meanings of participants.[40] During analysis, data were read thoroughly to aid familiarisation and coded to generate key themes. The themes were then examined and refined further. Sub-themes were subsequently developed, and categories representing similar themes were formed. A process of refinement continued until the final themes representing barriers and facilitators of medication adherence emerged.

Online forum- dataset

Methods are reported in greater detail elsewhere.[39] Briefly, the source of data was the archive file of an online forum, Talkstroke, hosted by the UK charity Stroke Association, between 2004 and

For peer review only

2011. We used thematic analysis informed by the PAPA theoretical framework, to analyse forum posts and derive themes on barriers and facilitators of adherence to secondary prevention medications.

Procedure and analysis

The literature was explored to identify characteristics associated with face to face and online forum approaches to qualitative data collection.[32 34 35 6 7] Key attributes that were considered to represent important aspects of each data source were identified and subsequently applied in the context of collecting/interpreting research data from each source.[36]. The key attributes were grouped into the three main domains Context, Mechanisms and Outcomes. Context included the attributes, location and sampling; Mechanisms included the attributes participation, dynamic of interaction, contribution, timing, guidance, communication; Outcomes included activities and reporting. See Table 1.

Key themes arising from the data were classified according to the Perception and Practicalities Approach (PAPA) [41] and interpreted according to the following two categories of the PAPA framework.

Perceptions: Necessity Beliefs and Concerns. Perceptual barriers and facilitators of medication adherence in stroke survivors and caregivers are explored within both sources of data, according to their classifications as necessity beliefs i.e. doubts about personal need for medication to maintain or improve current and future health, and concerns about secondary prevention treatment.

Practicalities- Capability and Resources. Barriers and facilitators that stroke survivors and caregivers face around their capability of taking/giving medication and the resources available to undertake such behaviour.

Table 1 here

Results

Details of participant characteristics are reported in Table 2. Of the 42 interviewed participants, 2/3 were stroke survivors and one third were caregivers. The median age of interviewed survivors was 72 years and the majority were female (21/28; 75%). The majority of interviewed caregivers were the stroke survivor's spouses. 64% of stroke survivors experienced a stroke within the previous 5 years, 22% in the last 12 months. 50% suffered from a stroke, predominantly ischaemic, 50% from a TIA. Interview participants were recruited from a single UK region.

Of the 84 online users, 58% were stroke survivors and 39% were caregivers. Forum survivors were on average aged 50 years. The median age of stroke survivors talked about by caregivers on the forum was 66 years, and 57% were female. 62% of caregivers in the online forum were daughters or sons, 28% spouses and the remainder were family members such as siblings or in-laws.90% of forum participants, who reported time since stroke, experienced it within the previous 5 years, 53% within the previous 12 months. It was not possible to determine the type of stroke experienced by users in the online forum.

A small number of participants in the forum were prolific, commenting frequently and offering encouragement to other participants. Forum users came from all over the UK.

Table 2 here

Despite the differences between the two sources of data as highlighted in Table 1, all key themes about barriers and facilitators to adherence to secondary prevention medications that emerged from the interview study could be matched with corresponding themes from the online forum (see Table 3).

For peer review only

The comparison of themes in the two data sources was facilitated by their previous classifications according to the PAPA framework. Comparison of themes in the interview and online forum studies are reported in Table 3.

Table 3 here

To understand attributes in the context of data source, we examined how they related to the themes identified within the two studies. Table 1 also shows the description of the two sources according to the data collection attributes identified.

The forum facilitated access to participants' views from across a wide geographical area compared to the views of a small group of survivors within a specific context (i.e. Eastern England GP practices). This may have limited interview participant's views, with the forum drawing on more varied and wide ranging healthcare setting experiences. The broader participants' sampling of the forum was reflected in the theme *'How seriously people take medicine for secondary prevention of stroke',* with online users being familiar with negative press on statins from across a variety of information sources, including research papers and online sources. Older interview participants recruited through GP practices instead might not be able to access this as easily, as reported in the '*Knowledge of stroke and medications'* theme. Within this theme, interviewed stroke survivors reported looking for medicine information in leaflets inside drug boxes, with forum participants seeking information mainly from resources such as healthcare professionals or online peers. Forum participants seemed keener to adopt a joint approach for medication taking with health professionals, which included patient-clinician shared decision-making about stopping medications.

Participation in the online forum meant that users had the possibility of taking part in multiple discussion threads. Despite the inability to ask clarification questions to participants of the forum, survivors and caregivers could read and reply to each other's' posts in an asynchronous way, with

For peer review only

online discussions allowing an in-depth exploration of themes about barriers and facilitators to secondary prevention medication taking. The '*ability to self-care'* theme is an example: survivor's perspective of handing over all responsibility of medications to caregivers was enriched by caregivers' posts describing how they were acting at times as advocates for stroke survivors with health professionals. The caregiver/survivor discussion dynamic among forum participants permitted conversations across and within patient and caregiver groups. Stroke survivors who were forum users offered advice and suggestions about medication as well as seeking reassurance and support. At the same time they were providing advice to caregivers on medicine taking or dealing with medication refusal, as reported in the themes on *regimen complexity and burden of treatment*.

The relative anonymity during online forum discussion and the absence of researcher's influence favoured openness among forum participants. For example, forum users were more likely to make frank admissions about decisions to refuse medicines, particularly statins, and about GP role in advising on medicines, as shown in the '*Taking medication'* theme. Discussions around this theme went as far as including clinicians' financial motivation behind prescriptions and questioning whether this was prioritised over health benefit. This contrasted with the dynamic reported by interview participants who seemed relying on GPs and were willing to do as the GP said.

Forum users had multiple opportunities for participation, with an open line of questioning guided by other survivors developing the conversation and widening the scope of the discussion. This contributed to data richness and important insights around the practicalities of medication taking, including difficult experiences with practical aspects of '*taking medications'* such as experiencing 'swallow panic'. The collaborative discussions between survivors and caregivers on the forum meant that users were more likely to offer each other practical medication taking strategies such as using a whiteboard. Caregivers in the online forum could communicate with other online caregivers separately from stroke survivors, manifesting their own opinions and attitudes towards secondary prevention medications. This did not emerge from interviews, when caregivers and survivors were interviewed together.

For peer review only

Discussion

In this analysis, themes that emerged from an interview study with stroke survivors and their caregivers could be matched with corresponding themes from users of an online stroke forum. This was true despite key differences in the attributes of data collection and the lack of verification of participants' identity and stroke diagnosis. An online stroke forum can be considered a valid source of data for qualitative research on patients' and caregivers' issues with medications after stroke. Perhaps because of the inclusion of a younger and computer literate population and the opportunity of online discussions between survivors and caregivers, forum data offered additional insights such as the effect of bad press on taking medicines, issues about clinician prescribing, and caregivers' reflections on their caregiving role, which may be outside the reach of a traditional qualitative sources of data like interviews.

Strengths and Limitations

This is the first investigation in stroke research to compare results from two independent studies addressing the same research question using two different data sources, a traditional one (interviews) and a novel one (online forum), according to the PAPA framework. The results suggest that qualitative studies on online stroke forums are strong and represent a step towards establishing the validity of an online stroke forum as source of qualitative research data.

A further strength of these results consists in highlighting characteristics that could be used to decide which source of data is more suitable to a particular research question, e.g. an online stroke forum could be more suitable when the focus is on gathering qualitative data from young computer-literate stroke survivors and young caregivers (most forum caregivers are sons and daughters of stroke survivors). With online comments provided directly from participants, the potential for ambiguity or distortion of patient views through transcription is reduced.

This investigation suggests that there is potential for an online forum to add depth to understanding of stroke survivors' issues with medications.

For peer review only

A limitation of this study is the inability to compare data from the two sources in respect to participants' characteristics like type of stroke, social class and geographic location. Body language, facial expressions and face to face interaction that were present in interviews cannot be compared with forum data. Third party moderation of an online forum raises the possibility of an online discussion being ended prematurely and posts excluded before they are analysed and therefore themes being missed.

While interviewed participants contributed to one or more themes and were included in the interview study, several forum participants mentioning secondary prevention medications were excluded from the forum study because they did not provide enough details to allow the identification of a theme. Despite this, because of the wealth of information shared online and the high number of forum participants, the exclusion of several participants did not affect data collection within the forum study.

Comparisons with existing research

In agreement with our findings, investigations of online versus face to face focus group discussions concluded that both methods could be used to answer research questions, that online forum is more suited to communicate opinions and capturing participants' perspectives from a wide geographical area, [34] and through anonymity discussions of more personal issues. [43]

Issues around the potential reliability of forum data in answering the research question may also arise out of concerns about whether the data are skewed toward a specific participant group. As the use of the Internet to conduct behavioural research grows, the representativeness of participants' samples and issues will remain challenging, in spite of the advantages of increased accessibility to otherwise hidden populations.[20]

An online forum as source of data collection offers the opportunity for cross communication and shared support among participants. Through this forum, users can also draw on a personalised support system based on peer experiences and built trust. [44] At the same time, as shown in this study, online cross communication between participants can enhance understanding and add

For peer review only

depth to the themes in qualitative research. With evidence that trust forms and develops on the online forum [45], there is the potential for this methodology to become an accepted and valued source of health information.[46]

Use of an online forum as a data collection technique in health care research raises potential ethical concerns around anonymity, privacy, confidentiality and informed consent compared to the more traditional qualitative approaches such as semi-structured interviews. [47 48]

Compared with face to face interviews where stroke survivors and caregivers were interviewed together, the forum offered a more neutral environment in which caregivers had the freedom to participate on their own. In this context, participants may be more willing to express deeply held personal opinions and to discuss sensitive issues more freely, as described by Allen and colleagues.[5] The knowledge of such issues has the potential to inform and improve involvement of both patients and their caregivers in the decision making process, thus facilitating a collaborative approach around the use of medication, and encouraging effective medication taking behaviour.[49] An interesting observation was that interview survivors were more likely to follow GPs' instructions around medicines whereas forum survivors reported a shared approach to decisions. This identifies an interesting dynamic around how older and younger survivors view the practitioner role. Indeed previous research confirms that older patients look to the GP for support and view the practitioner as trustworthy and an ally in making healthcare decisions. [50]

Implications for research

The online forum represents a source of data collection suited to capturing the views of a younger stroke population who have access to online resources and to information from press outlets, which can potentially influence their attitudes to medication. The presence of younger patients and caregivers in online stroke fora offer insight for the development of interventions targeted to these groups. Indeed, research has shown that those who are younger with poorer mobility report most unmet needs, including in respect of medication taking.[51 52]

For peer review only

Stroke survivors who struggle with face to face communication but can communicate using technology such as a computer or mobile phone can provide insight on their needs, informing clinical interventions designed to improve medication taking in this patient group.

In agreement with the work on cancer forums, our work shows that the potential of online communities as a source of data is only beginning to be realised. This comparison study suggests we can more confident in using data from online forums. Online forum data also offered unprecedented access to the caregiver perspective and the dynamic of their relationship with the stroke survivor and other with respect to barriers and facilitators to adherence to secondary prevention medications for stroke.

Conclusion

Both interviews and online forums are rich and useful sources of data and knowledge, revealing similar issues about patients' core experience. In uncovering additional themes the online forum may represent an important adjunct to traditional qualitative data collection methodologies.

Acknowledgements

We thank Jenni Burt and Chantal Balasooriya-Smeekens for their helpful comments on the manuscript.

Competing interests

None declared

Data sharing statement

No additional data are available

Author/s contribution

JJ contributed to the study design, data collection, data analysis and prepared the manuscript for submission. JM is a co-investigator on the study and commented on the manuscript. SS is a co-investigator on the study and commented on the manuscript. ADS contributed to the study design,

For peer review only

data analysis and commented on the manuscript. All authors agreed on the final draft of the submitted manuscript.

References

1. nesta.org.uk. Peer Support: What is it and does it work? Summarising evidence from more than 1000 studies. : Nesta, 2015.

2. Why I turned to social media for help after a double lung transplant. Online health forums are increasingly used as a way of getting peer support and taking control of an illness. *The Guardian* 2016.

3. Pendry LF, Salvatore J. Individual and social benefits of online discussion forums. *Computers in Human Behavior* 2015;50:211-20. doi: http://dx.doi.org/10.1016/j.chb.2015.03.067

4. Ennis–O'Connor. How online patient communities are changing the face of cancer care. In: Blog B, ed., 2014.

5. Allen C VI, Kennedy A, Rogers A. Long-Term Condition Self-Management Support in Online Communities: A Meta-Synthesis of Qualitative Papers. *J Med Internet Res* 2016;18(3):e61. doi: 10.2196/jmir.5260

6. Im E-O, Chee W. Practical Guidelines for Qualitative Research Using Online Forums. *Computers, informatics, nursing : CIN* 2012;30(11):604-11. doi: 10.1097/NXN.0b013e318266cade

7. Im E-O, Chee W. An Online Forum As a Qualitative Research Method: Practical Issues. *Nursing research* 2006;55(4):267-73.

8. De Simoni A, Shanks A, Mant J, et al. Making sense of patients' internet forums: a systematic method using discourse analysis. *British Journal of General Practice* 2014;64(620):e178-e80. doi: 10.3399/bjgp14X677671

9. Balasooriya-Smeekens C, Bateman A, Mant J, et al. Barriers and facilitators to staying in work after stroke: insight from an online forum. *BMJ Open* 2016;6(4) doi: 10.1136/bmjopen-2015-009974

For peer review only

10. Statistics OfN. Internet users in the UK: 2016 2016 [Available from: https://www.ons.gov.uk/businessindustryandtrade/itandinternetindustry/bulletins/internetusers/2016.

11. Ofcom. Internet use and Attitudes. Internet use and attitudes bulletin 2016, 2016.

12. Fox S. The Social Life of Health Information: Pew Research Centre; 2011 [Available from: http://www.pewinternet.org/2011/05/12/the-social-life-of-health-information-2011/.

13. Diaz JA, Griffith RA, Ng JJ, et al. Patients' Use of the Internet for Medical Information. *Journal of General Internal Medicine* 2002;17(3):180-85. doi: 10.1046/j.1525-1497.2002.10603.x

14. Ayers SL, Kronenfeld JJ. Chronic illness and health-seeking information on the Internet. *Health* 2007;11(3):327-47. doi: doi:10.1177/1363459307077547

15. McMullan M. Patients using the Internet to obtain health information: How this affects the patient–health professional relationship. *Patient Education and Counseling* 2006;63(1):24-28. doi: http://dx.doi.org/10.1016/j.pec.2005.10.006

16. Larner AJ. Searching the Internet for medical information: frequency over time and by age and gender in an outpatient population in the UK. *Journal of Telemedicine and Telecare* 2006;12(4):186-88. doi: doi:10.1258/135763306777488816

17. Andreassen HK, Bujnowska-Fedak MM, Chronaki CE, et al. European citizens' use of E-health services: A study of seven countries. *BMC Public Health* 2007 (Apr);10(7):53. doi: 10.1186/1471-2458-7-53

18. Taylor K. Connected health: How digital technology is transforming health and social care. *Deloitte Centre for Health Solutions* 2015

19. Moorhead SA, Hazlett DE, Harrison L, et al. A New Dimension of Health Care: Systematic Review of the Uses, Benefits, and Limitations of Social Media for Health Communication. *Journal of Medical Internet Research* 2013;15(4):e85. doi: 10.2196/jmir.1933

20. Rhodes SD, Bowie DA, Hergenrather KC. Collecting behavioural data using the world wide web: considerations for researchers. *Journal of Epidemiology and Community Health* 2003;57(1):68-73. doi: 10.1136/jech.57.1.68

21. De Simoni A, Shanks A, Balasooriya-Smeekens C, et al. Stroke survivors and their families receive information and support on an individual basis from an online forum: descriptive

For peer review only

analysis of a population of 2348 patients and qualitative study of a sample of participants. *BMJ Open* 2016;6(4) doi: 10.1136/bmjopen-2015-010501

22. Kaufman S, Whitehead KA. Producing, ratifying, and resisting support in an online support forum. *Health (London)* 2016 (Feb) doi: 10.1177/1363459315628043

23. McKechnie V, Barker C, Stott J. The Effectiveness of an Internet Support Forum for Carers of People With Dementia: A Pre-Post Cohort Study. *J Med Internet Res* 2014;16(2):e68. doi: 10.2196/jmir.3166 [published Online First: 28.02.2014]

24. Malik S, Coulson NS. The Therapeutic Potential of the Internet: Exploring Self-Help Processes in an Internet Forum for Young People With Inflammatory Bowel Disease. *Gastroenterology Nursing* 2011;34(6):439-48. doi: 10.1097/SGA.0b013e318237a9ba

25. Alinia H, Moradi Tuchayi S, Farhangian ME, et al. Rosacea patients seeking advice: Qualitative analysis of patients' posts on a rosacea support forum. *Journal of Dermatological Treatment* 2016;27(2):99-102. doi: 10.3109/09546634.2015.1133881

26. Zhao J, Abrahamson K, Anderson JG, et al. Trust, empathy, social identity, and contribution of knowledge within patient online communities. *Behaviour & Information Technology* 2013;32(10):1041-48. doi: 10.1080/0144929X.2013.819529

27. Kenny AJ. Interaction in cyberspace: an online focus group. *Journal of Advanced Nursing* 2005;49(4):414-22. doi: 10.1111/j.1365-2648.2004.03305.x

28. Opdenakker R. Advantages and Disadvantages of Four Interview Techniques in Qualitative Research. *Forum: Qualitative Social Research* 2006;7(4)

29. Seymour WS. In the flesh or online? Exploring qualitative research methodologies. *Qualitative Research* 2001;1(2):147-68. doi: doi:10.1177/146879410100100203

30. Britten N. Qualitative research: Qualitative interviews in medical research. . *BMJ* 1995;311:251-53.

31. Souter C, Kinnear A, Kinnear M, et al. Optimisation of secondary prevention of stroke: a qualitative study of stroke patients' beliefs, concerns and difficulties with their medicines. *International Journal of Pharmacy Practice* 2014;22(6):424-32. doi: 10.1111/ijpp.12104

For peer review only

32. Seale C, Charteris-Black J, MacFarlane A, et al. Interviews and Internet Forums: A Comparison of Two Sources of Qualitative Data. *Qualitative Health Research* 2010;20(5):595-606. doi: doi:10.1177/1049732309354094

33. Synnot A, Hill S, Summers M, et al. Comparing Face-to-Face and Online Qualitative Research With People With Multiple Sclerosis. *Qualitative Health Research* 2014;24(3):431-38. doi: 10.1177/1049732314523840

34. Schneider SJ, Kerwin J, Frechtling J, et al. Characteristics of the Discussion in Online and Face-to-Face Focus Groups. *Social Science Computer Review* 2002;20(1):31-42. doi: 10.1177/089443930202000104

35. Eva María Murgado-Armenteros, Francisco José Torres-Ruiz and Manuela Vega-Zamora. Differences between Online and Face to Face Focus Groups, Viewed through Two Approaches *Journal of Theoretical and Applied Electronic Commerce Research* 2012;7(2):73-86.

36. Pawson R TN. Realistic Evaluation. London: Sage 1997.

37. Britten N. Qualitative interviews in healthcare. In: Pope C MN, ed. Qualitative research in health care. 2nd ed. London: BMJ Books 1999:pp11-19.

38. Jamison J, Graffy J, Mullis R, et al. Barriers to medication adherence for the secondary prevention of stroke: a qualitative interview study in primary care. *British Journal of General Practice* 2016 doi: 10.3399/bjgp16X685609

39. Jamison J, Sutton S, Mant J, De Simoni, A Barriers and facilitators to adherence to secondary stroke prevention medications after stroke: Analysis of survivors' and caregivers' views from an online stroke forum. *BMJOpen* 2017;7(7):e016814. . doi: 10.1136/bmjopen-2017-016814. [published Online First: July 16]

40. Braun V, Clarke V. Using thematic analysis in psychology. *Qualitative Research in Psychology* 2006;3(2):77-101. doi: 10.1191/1478088706qp063oa

41. Horne R. Compliance adherence and Concordance. In: Harding KTG, ed. Pharmacy Practice. London: Taylor & Franics 2001.

42. Horne R WJ, Barber N, Elliott R, Morgan M. Concordance, adherence and compliance in medicine – taking. In: R&D RftNC-oCfNSDaO, ed., 2005.

For peer review only

43. Zwaanswijk M, van Dulmen S. Advantages of asynchronous online focus groups and face-to-face focus groups as perceived by child, adolescent and adult participants: a survey study. *BMC Research Notes* 2014;7:756. doi: 10.1186/1756-0500-7-756

44. Brady E, Segar J, Sanders C. “You get to know the people and whether they’re talking sense or not”: Negotiating trust on health-related forums. *Social Science & Medicine (1982)* 2016;162:151-57. doi: 10.1016/j.socscimed.2016.06.029

45. Gill PS, Whisnant B. A qualitative assessment of an online support community for ovarian cancer patients. *Patient Related Outcome Measures* 2012;3:51-58. doi: 10.2147/PROM.S36034

46. Cole J, Watkins C, Kleine D. Health Advice from Internet Discussion Forums: How Bad Is Dangerous? *Journal of Medical Internet Research* 2016;18(1):e4. doi: 10.2196/jmir.5051

47. Eysenbach G, Till JE. Ethical issues in qualitative research on internet communities. *BMJ* 2001;323(7321):1103-05. doi: 10.1136/bmj.323.7321.1103

48. British Psychological Society. Ethics Guidelines for Internet-mediated research. Leicester, 2013.

49. Belcher VN, Fried TR, Agostini JV, et al. Views of older adults on patient participation in medication-related decision making. *Journal of General Internal Medicine* 2006;21(4):298-303. doi: 10.1111/j.1525-1497.2006.00329.x

50. Butterworth JE, Campbell JL. Older patients and their GPs: shared decision making in enhancing trust. *The British Journal of General Practice* 2014;64(628):e709-e18. doi: 10.3399/bjgp14X682297

51. Jamison, J., di Tanna, G., Mant, J., Sutton, S., De Simoni, A.Evaluating the support stroke survivors get with daily medication taking in the community: interim results of a primary care survey. SAPC 2017; Warwick. EP1E.01.

52. Kersten P, Low JTS, Ashburn A, et al. The unmet needs of young people who have had a stroke: results of a national UK survey. *Disability and Rehabilitation* 2002;24(16):860-66. doi: 10.1080/09638280210142167

53. Bowker N, Tuffin K. Using the Online Medium for Discursive Research About People With Disabilities. *Social Science Computer Review* 2004;22(2):228-41. doi: doi:10.1177/0894439303262561

For peer review only

54. Mann C, & Stewart, F. Internet communication and qualitative research. Thousand Oaks, CA: Sage 2000.

55. Suler J. The Online Disinhibition Effect. *CyberPsychology & Behavior* 2004;7(3):321-26. doi: 10.1089/1094931041291295

56. DiCicco-Bloom B, Crabtree BF. The qualitative research interview. *Medical Education* 2006;40(4):314-21. doi: 10.1111/j.1365-2929.2006.02418.x

57. Stewart K, Williams M. Researching online populations: the use of online focus groups for social research. *Qualitative Research* 2005;5(4):395-416. doi: doi:10.1177/1468794105056916

58. Tanis M. Health-Related On-Line Forums: What's the Big Attraction? *Journal of Health Communication* 2008;13(7):698-714. doi: 10.1080/10810730802415316

59. White M, Dorman SM. Receiving social support online: implications for health education. *Health Education Research* 2001;16(6):693-707. doi: 10.1093/her/16.6.693

60. MacLean LM, Meyer M, Estable A. Improving Accuracy of Transcripts in Qualitative Research. *Qualitative Health Research* 2004;14(1):113-23. doi: doi:10.1177/1049732303259804

61. Wise K, Hamman B, Thorson K. Moderation, Response Rate, and Message Interactivity: Features of Online Communities and Their Effects on Intent to Participate. *Journal of Computer-Mediated Communication* 2006;12(1):24-41. doi: 10.1111/j.1083-6101.2006.00313.x

For peer review only

	Attribute	Online forum<br>*Literature (shaded)*<br>*Versus*<br>*Present study*	Semi-structured interviews<br>*Literature (shaded)*<br>*Versus*<br>*Present study*
**Context**	**Location**	Respondents from across a wide geographical area, can participate at own convenience.[53]	Interviewees geographically restricted.
		Patients' own home: UK	Patient's own home: East of England
	**Sampling**	Voluntary participation/ self-selection. Recruitment does not require collaboration between clinical sites or support of professional staff. [54]	Purposive recruitment in health care settings, guided by sampling techniques based on population demographics including age, gender and disability.
		Voluntary self-selection by participant: no sampling criteria, no restriction on age.<br>Verification of stroke/TIA diagnosis not possible.<br>Most participants taking part within the first 5 years since the cerebrovascular event	Purposive sampling: GP screened, pre-defined criteria to achieve maximum spread of gender and disability.<br>Age: 55 years and over. Confirmed stroke/TIA diagnosis
**Mechanisms**	**Participation**		
		Multiple participants per conversation thread: stroke survivors or caregivers.<br>Conversation possible between survivors, survivors and caregivers, or caregivers with other caregivers	Max 2 or 3 participants per single interview conversation: Researcher, stroke survivor and caregiver
	**Dynamic of interaction**	Discussion conducted remotely. Relative anonymity can encourage users to feel uninhibited.[5 55]<br>Likelihood of expressing honest opinions about sensitive issues.	Engagement can be actively encouraged.<br>Face to face approach enables development of rapport between interviewer and participant.[56]
		Less knowledge of participants, participants remain anonymous. No influence of researcher on participation.	Researcher's gain knowledge of interviewees, development of researcher-participant rapport, active encouragement of participation
	**Response contribution**	Longer conversations allow for a broader understanding of the subject matter and potential for greater depth.[57] Discussion threads generate reflection and greater description among users.[33]	Probing questions from researcher seeking clarification or to pursue a more detailed response.[37]
		Potential for significant individual contribution.<br>Responses shaped by other peer contributors.<br>More opportunity for self-reflection.<br>More frequent comments.	Maximum of two individual contributions (patients and caregiver).<br>Responses shaped by researcher interaction.<br>Less opportunity for self-reflection<br>In depth comments encouraged by researcher.

	**Timing of event**	Users can post repeatedly and frequently on many topics over a long period of time.	Interview is a single event occurring at one point in time Maximum of 2 contributors to the interview discussion.
		Single or multiple participation over time. Ability to contribute to discussion on multiple occasions/topics.	Single participatory event. Contribution fixed to a single time period.
	**Guidance**	User freedom to choose what to discuss, and how frequently to contribute to free-flowing discussion threads.[5]<br>Posts created through peer to peer communication, without professionals' involvement and influence.[58]<br>Response shaped by contribution of other survivors or caregivers.	Follows a pre-defined line of questioning. Several key questions define the area to be explored.[37]<br>Researcher oversees the direction of conversation.
		Free or peer-guided discussions.	Guided conversation: responses to pre-defined questions in topic guide
	**Communication**	Permits broad accessibility and asynchronicity with online communication.[5, 59]<br>Restricted to those with Internet access.[34]	Direct face to face, synchronous communication
		Indirect communication, via computer, no physical proximity, asynchronous	Direct communication, face to face, synchronous
**Outcomes**	**Activities**	No physical transcription is required; user contributions printed automatically, improving credibility of data.[7]<br>Potential for inaccurate interpretation through misunderstanding nuances in the data may still exist.[7]	Transcription is key to representing the individual and dependability of data. Transcription opens data to misinterpretation or misunderstanding.[60]<br>Rigour and accuracy in transcribing is integral to the analysis process, influencing the degree of dependability of data.[60]
		No audio recording.<br>Automatic transcription printed directly from forum<br>No field notes.	Interviews are audio recorded and interviews are transcribed. Potential for ambiguity through inaccurate transcribing<br>Field notes taken during interviews
	**Reporting**	Forum posts are moderated before appearing online, effect on the data collected is relatively unknown. Moderation processes can influence engagement in online communities.[61]	
		3rd party moderation leading to possible exclusion of data	No exclusion of data prior to analysis

Table 1. Key differences between an online forum approach and an interview approach to data collection.

	Interviews	Online forum
**Sample characteristics**	**N [Median] (Range)**	**N [Median] (Range)**
Total participants	42	84
**Age**		
Survivor	[72] (61-93)	[50] (32-72)
*Caregiver	-	
**Gender**		
Male -Survivor	7	20
Female- Survivor	21	26
Not known - Survivor	-	3
*Caregiver of Male Survivor	4	20
*Caregiver of Female Survivor	10	12
Unknown gender and unknown identity	-	3
**Identity person posting**		
Stroke survivor	28	49
Caregiver	14	33
Not known	-	2
**Years since stroke**		
(0-12 mths)	4	37
(1-5 yrs)	14	25
(6-10 yrs)	6	4
(11-15 yrs)	2	2
(15+ yrs)	2	1
Unknown	-	15
**Type of stroke**		
TIA	14	-
Ischaemic stroke	13	-
Haemorrhagic stroke	1	-
**Caregiver identity**		
Daughter /son	2	20
Spouse	12	9
Other (/in law/ sister)	-	3
Unknown	-	1
**Number of posts about secondary prevention**		
		37 (1 participant)
		15 (1 participant)
		1 (44 participants)
		2 (19 participants)
		3 (6 participants)
Legend: * Refers to 'caregiver' in interviews and 'Patient talked about by caregiver' in the forum discussions		

Table 2. Characteristics of participants of the online forum and interview study

For peer review only

For peer review only

Perceptions				
**Key themes identified (interviews)**	**Interview quote^**	**Interview participant (Age, gender)**	**Forum quote***	**Forum participant♦ (Age, gender)**
	**Treatment necessity**		**Treatment necessity**	
**1. Knowledge of stroke and medications**	The importance of taking this exactly on time is trivial. I would probably survive for a week, if I didn't take the. For a month I'd probably survive. It would not make any difference in two days.	Male, 86 yrs, N.03, ischaemic stroke	A female survivor commented that it was better to take a few extra tablets from the GP than to experience a stroke. Tablets were provided to prevent a further stroke, and she stressed that they shouldn't be stopped except on professional advice	Female, age 51, age at stroke 51, N.17
	Whenever I've got a new pill or anything I'd read the instructions only because they've made a mistake before now, like for instance they gave me one which I''m allergic to… So I keep check of what I'm taking now.	Male, 80 yrs N. 04, TIA	A stroke survivor recalled being on 75mg of aspirin as well as beta blockers, however, his nephew who was a consultant surgeon, suggested that had he been taking warfarin instead of the aspirin he may not have suffered a second stroke	Male, age 67, age at stroke 55, N.82
			(Bad press influences attitudes to medicines) A female survivor read about the hype around statins and stated she still didn't have confidence in them. She had read a research paper on statins suggesting they only added an extra 9 months of life. Her mum had been taking statins for 14 years and this still didn't prevent her arteries from clogging up	*Female, age 56, age at stroke 56, N.66*
			(Bad press influences attitudes to medicines) A survivor who had suffered 2 mini-strokes and been prescribed aspirin and cholesterol lowering statin but was refusing to take them because she had read in the press about bad side effects that they caused	Female, age unknown, age at stroke unknown, N.74
**2. Doubts about medicines**	I think aspirins are good for you. That's the one I fancy. Well it thins	Male, 75yrs, N.24, ischaemic	A survivor acknowledged statins were used to control cholesterol, but questioned whether high cholesterol	Male, age 67, age at stroke 55, n=70

For peer review only - http://bmjopen.bmj.com/site/about/guidelines.xhtml

	the blood and the blood it flows and that stops any clots so I do like to take it. I just don't see why I'm taking the other medication. I'm not fat or anything like that. I don't get very high blood pressure and well cholesterol, what is cholesterol..?	stroke	was actually a problem. He believed strokes occurred frequently, regardless of cholesterol levels. He talked about the 'Cholesterol Myth' having researched the topic on the Internet. He said he was feeling confused about the value of statins and taking these when in reality they weren't needed	
**3. Realisation of the importance of medicines**	At one time I wouldn't take a pill, I wouldn't even take an aspirin. Now I take it because I understand it keeps me alive. I just think it's fate, that's the way I look at it. If I stop taking medication I might as well lie down in the fast lane.	Male, 67yrs, N.12, ischaemic stroke	A male survivor already suffered 2 strokes and said it was impossible to ever fully recover from the experience. He said after his first stroke he was prescribed tablets he didn't take and he realizes this was a big mistake.	Male, age 67, age at stroke 55, n=82
Differing attitudes to medicines	Well I don't know what I'd be without taking them put it that way…because I've had a stroke and I've been fortunate. They keep me going, keep me on the straight and narrow.	Male, 73 yrs, N.11 ischaemic stroke	A female survivor felt it was better to take tablets from the GP than to experience another stroke. Tablets were provided to prevent another stroke and shouldn't be stopped except on professional advice	Female, age 51, age at stroke 51, N.17
	I refused it and I said well it's not because its rat poison. If you tell	Female, 71yrs, N.22	Another survivor remarked that, although being on pills was an inconvenience and she had stopped some	Female, age 52, age at stroke 52, N.76

For peer review only - http://bmjopen.bmj.com/site/about/guidelines.xhtml

	me I've got warfarin, I must be ill but if I take aspirin I can't be that ill.	TIA	medication, she continued to take aspirin and statin which she considered important	
	The only thing I like, I think aspirin's good for you, that's the only one I fancy. Well it thins the blood, and well thinning the blood makes it flow better and that stops any clots so I do like, like to take it	Male, 75 yrs N.25 ischaemic stroke	A survivor had suffered 2 strokes in the previous year, but none since commencing warfarin. She felt reassured by taking warfarin and worried about coming off the medication	Female, age 42, age at stroke 42, N.35
	I just take them because the hospital prescribed them. If the doctor prescribed them I probably wouldn't bother. I'd probably say forget about it. He's a consultant so he should know what he's talking about	Male, 67 yrs N.15 ischaemic stroke	A survivor described how he trusted his vascular surgeon who had changed his medication from warfarin to aspirin and statin. The survivor was happy to take aspirin and felt it would be good to continue as the surgeon also took it regularly, concluding it must be beneficial and would enable him to live longer.	Male, age 35, age at stroke 34, N.71
			A female survivor decided to reduce cholesterol using diet instead, because of side effects from medication. She felt that once the symptoms completely disappeared she wouldn't take a statin again. She said she would start taking olive oil and follow a healthy diet to keep her cholesterol balanced naturally. She said she would continue aspirin as it didn't seem to cause any side effects	Female, age 52, age at stroke 52, N.76
	**Treatment Concerns**		**Treatment Concerns**	
**4. How seriously people take medicines for secondary prevention of stroke**	I wouldn't take them because I still, to me, blood pressure and cholesterol tablets to me I don't see what they're doing for me	Male, 75 yrs, N.24, ischaemic stroke	A female survivor who had read bad reports about statins reported being nervous about them. She didn't want to jeopardise feeling good by taking medication that she wasn't convinced she needed	Female, age 54, age at stroke 54, N.37

For peer review only

	Well she gave me blood pressure pills and that but I didn't take them. I felt so, i didn't bother, pity really. But never mind. I do now. I'm religious about that. I'll have another stroke if I don't. Didn't want to put the family through that again.	Male, 65 yrs, N.02, ischaemic stroke	A survivor refused statins after her first stroke because of side effects. However, after suffering a second one she was now worried enough to take them.	Survivor, female, age 68, age at stroke 67, N.14
**5. Taking medications**<br>*Nonadherence to medicine*	Well now and again I forget the cholesterol because that's the one at night and it's the only one I take at night	Male, 67yrs, N.15 ischaemic stroke	A male survivor said he was on 2 tablets for blood pressure and that he continued to take one every day. But the other was a diuretic and having got fed up frequently running to the toilet, he decided to check his blood pressure every day and would skip the diuretic if it was fine.	Male, age unknown, age at stroke unknown, N.63
Trust in GP	So if the doctor says take ten pills a day, I'll, I'll do it….he makes the decision and erm he, he's the boss man as you might say, who knows what he's up to<br><br>I do exactly as the Dr tells me. He's the Dr isn't he. He should know better than what I do. I don't push them anymore and say well you know I don't like taking this	Male, 87yrs, N.8, TIA<br><br>Male, 80 yrs N.04, TIA	*A male survivor agreed with his doctor to stop taking a blood pressure tablet because of intolerable side effects, and his wife being a nurse made it easier. He felt strongly that doctors are there to advise not instruct.*<br><br>*(Collaborating with GP/ patient)*<br>*A caregiver said that her husband ceased taking medications except aspirin, because of side effects. He made this decision, together with the GP and stressed the importance of doing this before stopping tablets*	*Male, age unknown, age at stroke unknown, N.63*<br><br>Male, age 54, age at stroke 52, N.68

For peer review only - http://bmjopen.bmj.com/site/about/guidelines.xhtml

			Concern<br>*(Prescribing concerns)*<br>*A caregiver (sister) suggested that GPs shouldn't be paid for prescribing statins with the decision based on clinical judgement alone. The involvement of money could lead to medication being over prescribed for financial reasons*<br>*Concern*<br>*(Prescribing concerns)*<br>*A survivor described taking 9 pills a day for stroke and its side effects and felt that the GP should understand which were necessary. Following another appointment her consultant was furious about the medications she had been prescribed.*	Gender and age unknown, age at stroke unknown, N.78<br><br>Female, age 37, age at stroke 36
**Practicalities**				
	**Capability/ resources**		**Capability/ resources**	
**6. Ability to self-care**	My wife sorts it out and that's why I don't know so much about it you see she [taps].She puts them there, I take them and that's it	Male, 80 yrs, N.04, TIA	A caregiver stated that she was providing the stroke survivor with all of his medication due to his poor memory as a result of the stroke. She was now in complete control of his medication which she was happy about but it was difficult as he was a loved one and something she had no training for.<br><br>*(Caregiver as an advocate for the stroke survivor)*<br>*A female caregiver described consistently trying to have her husband's 40mg statin dosage reduced by his GP. As a result of the high dosage he was chronically tired, so he stopped taking statins*<br><br>*(Caregiver as an advocate for the stroke survivor)*<br>*A caregiver recommended being firm with GPs about being prescribed atorvastatin if simvastatin was not*	Female, Age 46, age at stroke 40, N.5<br><br>Male, age 54, age at stroke 52, N.68<br><br>Gender and age unknown, age at stroke unknown,

For peer review only - http://bmjopen.bmj.com/site/about/guidelines.xhtml

			tolerated, as atorvastatin was a bit more expensive but recommended by NICE guidelines as an alternative	N.18
**7. Taking medication**<br><br>*Problems swallowing*<br><br>*Accessing packaged medicines*	The big ones, I, do actually feel I have to swallow two or three times to get them down<br><br>Some of the, the pills are a hell of a trouble, you know the bubble wrap, flipping them out especially with my hands not as strong as they should be<br><br>I'd have to rely on the wife to…cause I can't get them out the packet, just can't get your hands in	Male, 66yrs, N.10 TIA<br><br>Male, 87yrs, N.08, TIA<br><br>Male, 65yrs, N.02 Ischaemic stroke	A male survivor described 'swallow panic', i.e. fear of choking when trying to take Dipyridamol capsules. The user said it took around 3 months before he got over that.<br><br>A caregiver (son in law) mentioned that despite the use of a nomad tray, tablets were still being taken from the wrong day with several days tablets being taken in a single day. The stroke survivor often didn't take the time to work out the days or to look at the calendar.<br><br>A survivor agreed with another user about the problem with the size of dipyridamole tablets, which were getting stuck in the pill box organizer.	Male, age unknown, age at stroke unknown, n=85<br><br>Male, age unknown, age at stroke unknown, n=111<br><br>Female, age 46, age at stroke 45, N.30
**8. Medication routines**	I only remember to take the others if I take them out of the cupboard the night before and leave them on the top. If I didn't I would probably forget.. because it isn't the first thing that I think of<br><br>I usually take it around 5 o'clock which strangely enough is about the time that we feed the dog and normally speaking I take the medication then I get his dinner	Male, 66yrs, N.10, TIA<br><br>Male, 68 yrs N.09 TIA	A female survivor described keeping the pill box in a specific location in the house, such as by the kettle, which then acted as a reminder to check the medication box<br><br>A survivor suggested using a white board and having method in place helped. She remembered taking her own medications through repetition or linking tablet use to another everyday activity	Female, age 60, age at stroke 60,<br><br>Female, age 54, age at stroke 46, N.19

For peer review only - http://bmjopen.bmj.com/site/about/guidelines.xhtml

**9. Changing medications**	They changed his medication to cheaper cholesterol and Dean was physically ill. He couldn't cope on it at all so he went back and the doctor said 'oh well it was just to try and they put him back on the others	Female, caregiver, N.24, age unknown	A survivor described being on 80 mg of simvastatin which they were happy with but that upon leaving hospital the dose was halved by the consultant which had very bad consequences, resulting in daily angina turns for a week. In the end he had to go back to his GP and be put back on the 80mg dose.	Female, age 53, age at stroke 50,
	She gave me an extra pill and I had a horrific night. She made an apology and said I'm sorry it took so long to get it right, but the fine tuning takes a bit of doing.	Male, 80 yrs N.04 TIA	A male survivor said he was taking up to 7 different blood pressure tablets and that it was unusual to only need a few tablets. He recommended going back to the GP as necessary to keep changing tablets until the correct combination was found.	Male, age 52, age at stroke 52, N.64
**10. Regimen complexity and burden of treatment**	I have to take 10 a day now altogether but I went up there (to the practice) to say can I get off some of these tablets, and I come back and I was on an extra one so I've not been up since	Male, 70yrs, N.13, TIA	A caregiver (son) was asking advice on how to encourage medication taking. His mother was originally taking multiple tablets up to 4 times a day but that now she was refusing to take them all and he was upset by this. Persuading her to continue taking the most important tablets had taken hours to do.	Female, age 77, age at stroke 77, N.9
	I've got yards of them. I don't know half the names I'm just told when to take them. That's one thing I'd like to do away with	Male, 73 yrs, N.11, Ischaemic stroke	A caregiver (wife) described how her husband was adamant he was not prepared to take statins because he didn't have the time to keep going back to the GP for checkups. The caregiver said she was feeling helpless and wasn't sure what she could do about it	Male, age 55, age at stroke 55, N.24
			(Burden of side effects on stroke survivor) A survivor described similar side effects from 3 different statins despite varying the medication dosage. She said tests confirmed this and she concluded long term use could result in problems that had a negative effect on her quality of life.	Female, age 34yrs, age at stroke 32, N.36
*Quote not transcribed - described to protect user confidentiality.				

For peer review only - http://bmjopen.bmj.com/site/about/guidelines.xhtml

^Quote transcribed<br>♦ Demographic characteristics relate to the stroke survivors only (either talking in first person or talked about by a caregiver).<br>Quotes in italics refer to additional themes identified in the online forum only

Table 3. Comparison of themes identified through analysis of semi-structured interviews and the online forum

For peer review only

For peer review only - http://bmjopen.bmj.com/site/about/guidelines.xhtml

Consolidated criteria for reporting qualitative studies (COREQ): 32-item checklist

No	Item	Guide questions/description	
**Domain 1: Research team and reflexivity**			
Personal Characteristics			
1.	Interviewer/facilitator	Which author/s conducted the interview or focus group?	N/A
2.	Credentials	What were the researcher's credentials? *E.g. PhD, MD*	BSc, MSc
3.	Occupation	What was their occupation at the time of the study?	Research Assistant/ PhD Student
4.	Gender	Was the researcher male or female?	Male
5.	Experience and training	What experience or training did the researcher have?	JJ is an experienced researcher with expertise in qualitative research.
Relationship with participants			
6.	Relationship established	Was a relationship established prior to study commencement?	N/A
7.	Participant knowledge of the interviewer	What did the participants know about the researcher? e.g. *personal goals, reasons for doing the research*	N/A
8.	Interviewer characteristics	What characteristics were reported about the interviewer/facilitator? e.g. *Bias, assumptions, reasons and interests in the research topic*	N/A
**Domain 2: study design**			
Theoretical framework			
9.	Methodological orientation and Theory	What methodological orientation was stated to underpin the study? *e.g. grounded theory, discourse analysis,*	Realist Evaluation Perspective. PAPA (Perception and Practicalities Approach. **Pg 6 + 7**

For peer review only

No	Item	Guide questions/description	
		ethnography, phenomenology, content analysis	
Participant selection			
10.	Sampling	How were participants selected? *e.g. purposive, convenience, consecutive, snowball*	N/A
11.	Method of approach	How were participants approached? e.*g. face-to-face, telephone, mail, email*	N/A
12.	Sample size	How many participants were in the study?	N/A
13.	Non-participation	How many people refused to participate or dropped out? Reasons?	N/A
Setting			
14.	Setting of data collection	Where was the data collected? e.*g. home, clinic, workplace*	Forum: online web site. Interviews: in the patient/caregiver home. **Pg 6**
15.	Presence of non-participants	Was anyone else present besides the participants and researchers?	No
16.	Description of sample	What are the important characteristics of the sample? *e.g. demographic data, date*	Gender, age, stroke survivor/ caregiver, years since stroke occurred. **Table 2**
Data collection			
17.	Interview guide	Were questions, prompts, guides provided by the authors? Was it pilot tested?	N/A
18.	Repeat interviews	Were repeat interviews carried out? If yes, how many?	N/A
19.	Audio/visual recording	Did the research use audio or visual recording to collect the data?	N/A

For peer review only

**No**	**Item**	**Guide questions/description**	
20.	Field notes	Were field notes made during and/or after the interview or focus group?	N/A
21.	Duration	What was the duration of the interviews or focus group?	N/A
22.	Data saturation	Was data saturation discussed?	N/A
23.	Transcripts returned	Were transcripts returned to participants for comment and/or correction?	N/A
**Domain 3: analysis and findings**			
Data analysis			
24.	Number of data coders	How many data coders coded the data?	Interview: JJ-100%. **Pg. 6**
25.	Description of the coding tree	Did authors provide a description of the coding tree?	Table 3 reports themes identified and coded from the Interview/ Forum data. **Table 3.**
26.	Derivation of themes	Were themes identified in advance or derived from the data?	Themes were generated from the data. **Pg. 6**
27.	Software	What software, if applicable, was used to manage the data?	Microsoft Excel and Nvivo used in each of the included studies. **Pg. 6**
28.	Participant checking	Did participants provide feedback on the findings?	N/A
Reporting			
29.	Quotations presented	Were participant quotations presented to illustrate the themes / findings? Was each quotation identified? e.g. *participant number*	Forum: Yes, quotes presented as descriptions to protect confidentiality. Interview: Yes. Identification by unique participant number only. **Table 3**
30.	Data and findings consistent	Was there consistency between the data presented	Yes. **Table 3**

For peer review only

No	Item	Guide questions/description	
		and the findings?	
31.	Clarity of major themes	Were major themes clearly presented in the findings?	Yes. **Table 3**
32.	Clarity of minor themes	Is there a description of diverse cases or discussion of minor themes?	Yes. Key themes and sub-themes are reported. **Table 3**

For peer review only

BMJ Open

An online stroke forum as source of data for qualitative research: insights from a comparison with patients' interviews

Journal:	*BMJ Open*
Manuscript ID	bmjopen-2017-020133.R1
Article Type:	Research
Date Submitted by the Author:	19-Jan-2018
Complete List of Authors:	JAMISON, JAMES; UNIVERSITY OF CAMBRIDGE, PUBLIC HEALTH & PRIMARY CARE<br>Sutton, Stephen; University of Cambridge,<br>Mant, Jonathan; University of Cambridge, General Practice and Primary Care Research Unit<br>De Simoni, Anna; Queen Mary University of London, Centre for Primary Care and Public Health
**Primary Subject Heading**:	Research methods
Secondary Subject Heading:	Qualitative research, Research methods, Cardiovascular medicine
Keywords:	STROKE MEDICINE, QUALITATIVE RESEARCH, Online forum, Methodology

SCHOLARONE™
Manuscripts

An online stroke forum as source of data for qualitative research: insights from a comparison with patients' interviews

*James Jamison[1]

Stephen Sutton[1]

Jonathan Mant[1]

Anna De Simoni[2]

[1]Primary Care Unit, Institute of Public Health, Forvie Site, University of Cambridge School of Clinical Medicine, Box 113 Cambridge Biomedical Campus, Cambridge, CB2 0SR.

[2]Centre for Primary Care and Public Health, Barts and the London School of Medicine and Dentistry, Queen Mary University of London, London, E1 2AB.
Email: a.desimoni@qmul.ac.uk Phone: Tel +44 (0)207 882 2520

*corresponding author: James Jamison; Tel: +44 (0)1223 768272. Fax: +44 (0)1223 763492. Email: jj285@medschl.cam.ac.uk

For peer review only

Abstract

Objective

To determine the appropriateness of an online forum compared with face-to-face interviews as a source of data for qualitative research on adherence to secondary prevention medications after stroke.

Design

A comparison of attributes of two data sources, interviews and a forum, using realistic evaluation; a comparison of themes around adherence according to the Perceptions and Practicalities Approach (PAPA) framework.

Setting

Interviews were conducted in UK GP practices in 2013 and 2014; Online posts were written by UK stroke survivors and family members taking part in the online forum of the Stroke Association between 2004 and 2011.

Participants

42 interview participants: 28 stroke survivors (age range 61-92yrs), 14 caregivers (85% spouses). 84 online forum participants: 49 stroke survivors (age range 32-72yrs), 33 caregivers (60% sons/daughters).

Results

10 attributes were identified within the two data sources, and categorised under 3 domains (Context, Mechanisms and Outcomes). Participants' characteristics of forum users were often missing. Most forum participants had experienced a stroke within the previous 12 months, while interviewees had done so one to five years previously.

All interview themes could be matched with corresponding themes from the forum.The forum yielded three additional themes: influence of bad press on taking statins, criticisms of clinicians' prescribing practices, and caregiver burden in assisting with medications and being advocates for survivors with healthcare professionals.

For peer review only

Conclusions

An online forum is an appropriate source of data for qualitative research on patients' and caregivers' issues with adherence to secondary prevention stroke medications and may offer additional insights compared to interviews, which can be attributed to differences in the approach to data collection.

Abstract word count- 263

Keywords: Stroke, Online forum, Qualitative interviews, Methodology

Article summary

Strengths and Limitations

- Informed by the literature and using a realistic evaluation approach, this study provides a structured framework to systematically explore differences between two data collection approaches, which may be of use to other researchers.
- The differences in study participants and themes highlighted here could be used to inform researchers on which data collection approach would be most appropriate for the research question being asked.
- Understanding of themes was not affected by potential limitations of an online forum such as lack of knowledge of participant characteristics, absence of face to face interaction and inability to directly question participants.
- With no control over the direction of discussions, the effectiveness of the online forum as a source of data will depend on how well the forum posts address the research question.
- Younger forum participants were potentially more engaged with managing their condition than their offline interviewed counterparts, perhaps representing people with a good understanding of their health.

For peer review only

Funding: Anna De Simoni is funded by a NIHR Academic Clinical Lectureship. James Jamison was supported by a research grant from The Stroke Association and the British Heart Foundation: TSA BHF 2011/01

Data sharing statement: No additional data available.

Introduction

In recent years participation in online forums has increased dramatically out of a need for patients to know more about the health care conditions they face. [1 2 3 4 5] With the use of online health forums steadily increasing, greater efforts are being made to understand this mode of data collection for qualitative research. [6 7] Online patients' communities represent an important source of information offering access to hard-to-reach groups who are often excluded (or exclude themselves) from traditional research studies. [8 9]

Internet use across the UK has grown considerably. A recent report on Internet usage found that in the first quarter of 2017, approximately 89% of adults used the Internet. From 2011 to 2016 internet use has risen from 52% to 78% in 65 to 74 year olds.[10] 42% of those 75 years and older of all genders are now Internet users.[11] It is estimated that one in four people with a chronic condition who use the Internet go online to find others with similar health concerns.[12] Patients engage with the Internet to access health information [13] and manage chronic illness.[14]

Patients are becoming more informed about their health through using the Internet.[15 16] Around 70% of Europeans who access the Internet, using it to obtain health information.[17] In the UK, digital technology has recently emerged as a key vehicle for the delivery of health and social care. [18] A review of the use of technology in healthcare confirmed that social media were increasingly used to communicate health information among public, patients and health professionals. [19]

As a method of capturing data on health attitudes and behaviour, the online forum offers considerable advantages, including access to large numbers of prospective participants with the

For peer review only

potential for open and honest discussions.[20 8 9 21] Such forums have been used previously in health care research [22] across a range of health domains. [23 24 25 26]

In the face of increased technological change, there is a growing need to understand the potential for online sources of qualitative data [27] and their advantages and disadvantages compared with traditional data collection techniques.[28 29]

The face to face interview is an important qualitative data collection technique widely used in health care research. [30 31] The method permits close observation of respondents, flexibility to adapt the direction of conversation and the scrutiny of physical cues such as body language.

There is a growing body of literature exploring the potential for online forums, as a source of data collection compared with traditional qualitative techniques as well as a greater understanding around using each of these methods. Nevertheless, difficulties verifying participants' identity and medical condition (i.e. are forum users real patients with stroke?) as well as the inability to interpret visual cues and seek clarification to questions, suggest that it may be necessary to confirm results with more established data sources, such as qualitative interviews. Confirmation of data may be deemed necessary in order to explore whether forum findings are representative of issues experienced by patients with stroke, and whether differences between the two sources could be used to decide which is better suited to addressing a particular research question. In an investigation comparing an online forum with qualitative interviews among cancer patients, the authors concluded that the forum offered useful data for qualitative health research.[32] Similarly, comparison between an online forum and face to face focus groups in people with multiple sclerosis concluded that forum results were comparable. [33] Comparison of characteristics of online versus face to face approaches have been reported more frequently with respect to focus groups.[34 35]

Drawing on a realistic evaluation perspective, [36] the objective of the present study was to explore differences between two approaches to data collection, interviews[37] and an online stroke forum[38], by seeking to understand the attributes that underpin each data source, exploring the context within each data collection occur and comparing barriers and facilitators to adherence to secondary prevention medication classified thematically according to the PAPA[39] framework. Our overall aim was to offer a structured way to systematically explore the differences between these two data collection approaches

For peer review only

and highlight the characteristics of an online stroke forum as source of data for qualitative research, which may be of use to other researchers.

Methods

Design

Comparison of themes around barriers and facilitators of adherence to secondary prevention medications after stroke in two independent studies, qualitative semi-structured interviews and an online forum. Although one author (JJ) initially coded the data in both studies, a subset of each dataset was double coded by a different author in each of the studies, therefore thematic analyses were independently validated. As interviews are a widely accepted method in qualitative research, [40] we used this as the standard against which to compare forum data. Differences and similarities in the data were examined, and results were compared and contrasted to explore the potential of the online forum as a data collection source.

Interviews-dataset

Interview participants included stroke survivors recruited through five General practice surgeries in Eastern England, along with their caregivers, as described previously.[37] In brief, purposive sampling was undertaken, patients were approached by letter and positive responders were contacted to confirm attendance. All interviews were guided by a topic schedule, with written consent. They were conducted in the stroke survivors own houses together with caregivers and lasted approximately one hour. 20% of the interviews were double coded by another author to ensure rigour and strengthen the validity of findings. The interview study was granted ethical approval by NHS Research South Yorkshire Ethics Committee (Ref 13-YH-0067).

Online forum-dataset

Methods are reported in greater detail elsewhere.[38] Briefly, the source of data was the archive file of an online forum, Talkstroke, hosted by the UK charity Stroke Association, between

For peer review only

2004 and 2011. This was a moderated forum, set-up as part of the charity website with the scope of facilitating online communication between stroke survivors and caregivers, sharing information on about any aspect of stroke and offering emotional support.

Barriers and facilitators of adherence were identified through analysis of a set of pre-defined keywords related to secondary prevention and stroke. Forum posts were explored using thematic analysis. Key themes were developed, representing barriers and facilitators of medication adherence. As these themes were further refined, subthemes were identified and a coding framework was developed. Forum posts were coded to identify practical and perceptual factors affecting adherence to medication, guided by the PAPA framework. In the final stage of the analysis, themes that were identified were mapped onto the theory and then subdivided to represent barriers or facilitators of adherence. To ensure rigour, another researcher who was not involved in coding the interviews, double coded half of all the forum posts identified.

In respect to ethical approval, the stroke Association granted permission to use the stroke forum data for research purposes before analysis of the data commenced. Informed consent was not sought from forum participants although forum users were aware that by participating in a public forum, their responses were available for others to view online. Verbatim quotes posted in the online forum were not used to protect the identity and intellectual property of participants, despite this being normal practice in qualitative research, we only used descriptions of quotes throughout the text.[9 21] To minimise the risk of interpretation bias, we ensured the paraphrasing of text reflected as closely as possible to the original forum posts. The ethical aspects of conducting research on this forum have been discussed more extensively elsewhere. [21 41]

Procedure and analysis

To allow us to directly compare the analyses from these interviews and an online stroke forum [38], transcripts of the interview study were re-analysed in Nvivo 10 using a thematic analysis [42] according to the PAPA framework (see details in the section Procedure and Analysis).[39 43]

For peer review only

Comparison of attributes of the two data sources

The literature was explored by two authors (JJ and ADS) to isolate characteristics associated with face to face and online forum approaches to qualitative data collection.[32 34 35 6 7]The evidence was discussed with experts in qualitative research methods, and subsequently 10 attributes identified as representing the key characteristics of both methods of data collection. Attributes were categorised according to the domains of realistic evaluation – context, mechanisms and outcomes. The classification of attributes into context, mechanisms and outcomes was discussed until a final consensus was reached. The key attributes that were considered to represent important aspects of each data source were subsequently applied in the context of collecting/interpreting research data from each source.[36] Context included the attributes location and sampling; Mechanisms included the attributes participation, dynamic of interaction, contribution, timing, guidance, communication; Outcomes included activities and reporting. See Table 1.

Comparison of themes using PAPA thematic analysis

Key themes arising from the data were classified according to the Perception and Practicalities Approach (PAPA) [39] and interpreted according to the following two categories of the PAPA framework.

Perceptions: Necessity Beliefs and Concerns. Perceptual barriers and facilitators of medication adherence in stroke survivors and caregivers are explored within both sources of data, according to their classifications as necessity beliefs i.e. doubts about personal need for medication to maintain or improve current and future health, and concerns about secondary prevention treatment.

Practicalities- Capability and Resources. Barriers and facilitators that stroke survivors and caregivers face around their capability of taking/giving medication and the resources available to undertake such behaviour.

Table 1 here

For peer review only - http://bmjopen.bmj.com/site/about/guidelines.xhtml

Results

Details of participant characteristics are reported in Table 2. Of the 42 interviewed participants, 2/3 were stroke survivors and one third were caregivers. The median age of interviewed survivors was 72 years (Range: 61-92 yrs) and the majority were female (21/28; 75%). The majority of interviewed caregivers were the stroke survivor's spouses. 64% of stroke survivors experienced a stroke within the previous 5 years, 22% in the last 12 months. 50% suffered from a stroke, predominantly ischaemic, 50% from a TIA. Interview participants were recruited from a single UK region.

Of the 84 online users, 58% were stroke survivors and 39% were caregivers. Forum survivors were on average aged 50 years (Range: 32-72 yrs). The median age of stroke survivors talked about by caregivers on the forum was 66 years (Range 46-91 yrs), and 57% were female. 62% of caregivers in the online forum were daughters or sons, 28% spouses and the remainder was family members such as siblings or in-laws. 90% of forum participants, who reported time since stroke, experienced it within the previous 5 years, 53% within the previous 12 months. It was not possible to determine the type of stroke experienced by users in the online forum.

A small number of participants in the forum were prolific, commenting frequently and offering encouragement to other participants. Forum users came from all over the UK.

Table 2 here

Comparison of themes according to PAPA

Despite the differences between the two sources of data as highlighted in Table 1, all key themes about barriers and facilitators to adherence to secondary prevention medications that emerged from the interview study could be matched with corresponding themes from the online forum (see Table 3). The comparison of themes in the two data sources was facilitated by their classifications according to the PAPA framework and details are reported in Table 3.

Three additional themes were identified in the forum, which did not emerge from interviews. First, stroke survivors openly discussed the influence of bad press on medication taking, in particularly around statins. Second, forum users raised concerns around healthcare professionals' prescribing practices and financial incentives to prescribe. Third, caregivers' difficulties with ensuring adherence secondary prevention medications, and struggles acting as advocates for patients with healthcare professionals.

Table 3 here

Comparison of the two sources of data

To understand attributes in the context of data source, we examined how they related to the themes identified within the two studies. Table 1 also shows the description of the two sources according to the data collection attributes identified.

The forum facilitated access to participants' views from across a wide geographical area compared to the views of a small group of survivors within a specific context (i.e. Eastern England GP practices). This may have limited interview participant's views, with the forum drawing on more varied and wide ranging healthcare setting experiences.

The sampling of participants in the forum was reflected in the theme *'How seriously people take medicine for secondary prevention of stroke',* with online users being familiar with negative press on statins from across a variety of information sources, including research papers and online sources. Older interview participants recruited through GP practices instead might not be able to access this as easily, as reported in the '*Knowledge of stroke and medications'* theme. Within this theme, interviewed stroke survivors reported looking for medicine information in leaflets inside drug boxes, with forum participants seeking information mainly from resources such as healthcare professionals or online peers. Forum participants seemed keener to adopt a joint approach for medication taking with health professionals, which included patient-clinician shared decision-making about stopping medications.

For peer review only

Participation in the online forum meant that users had the possibility of taking part in multiple discussion threads. Despite the inability to ask clarification questions or to probe participants of the forum, survivors and caregivers could read and reply to each other's' posts in an asynchronous way, with online discussions allowing an in-depth exploration of themes about barriers and facilitators to secondary prevention medication taking. The '*ability to self-care'* theme is an example: survivor's perspective of handing over all responsibility of medications to caregivers was enriched by caregivers' posts describing how they were acting at times as advocates for stroke survivors with health professionals. As has been highlighted in previous research in the field [33], the caregiver/survivor discussion dynamic among forum participants permitted conversations across and within patient and caregiver groups. Stroke survivors who were forum users offered advice and suggestions about medication as well as seeking reassurance and support. At the same time they were providing advice to caregivers on medicine taking or dealing with medication refusal, as reported in the themes on *regimen complexity and burden of treatment*.

The relative anonymity during online forum discussion and the absence of researcher's influence favoured openness among forum participants. For example, forum users were more likely to make frank admissions about decisions to refuse medicines, particularly statins, and about GP role in advising on medicines, as shown in the '*Taking medication'* theme. While the younger age of forum participants may have contributed to this, interviews with stroke survivors were conducted in the presence of the caregiver and this may have encouraged a level of self-censorship. Discussions around this theme went as far as including clinicians' financial motivation behind prescriptions and questioning whether this was prioritised over health benefit. This contrasted with the dynamic reported by interview participants who seemed relying on GPs and were willing to do as the GP said.

Forum users had multiple opportunities for participation, with an open line of questioning guided by other survivors developing the conversation and widening the scope of the discussion. This contributed to data richness and important insights around the practicalities of medication taking, including difficult experiences with practical aspects of '*taking medications'* such as experiencing 'swallow panic'. The collaborative discussions between survivors and caregivers on the forum meant that users were likely to offer each other practical medication taking strategies such as using a

whiteboard. Caregivers in the online forum could communicate with other online caregivers separately from stroke survivors, manifesting their own opinions and attitudes towards secondary prevention medications. This did not emerge from interviews, when caregivers and survivors were interviewed together.

Discussion

In this analysis, themes that emerged from an interview study with stroke survivors and their caregivers could be matched with corresponding themes from users of an online stroke forum. This was true despite key differences in the attributes of data collection and the lack of verification of participants' identity and stroke diagnosis. An online stroke forum can be considered a trustworthy source of data for qualitative research on patients' and caregivers' issues with medications after stroke. Perhaps because of the inclusion of a younger and computer literate population and the opportunity of online discussions between survivors and caregivers, forum data offered additional insights such as the effect of bad press on taking medicines, issues about clinician prescribing, and easy access to caregivers' reflections on their caregiving role.

Strengths and Limitations

This investigation compares results from two studies addressing the same research question using two different data sources, a traditional one (interviews) and a novel one (online forum), according to the PAPA framework. The results suggest that qualitative studies on online stroke forums are strong and represent a step towards confirming that an online stroke forum is a trustworthy source of qualitative research data. A further strength is the development and use of a structured framework informed by previous literature, identifying important differences in the attributes of each of the methods of data collection. Furthermore, these results highlight characteristics that researchers could use to decide which source of data is more suitable to a particular research question, e.g. an online stroke forum could be more suitable when the focus is on gathering qualitative data from

For peer review only

young computer-literate stroke survivors and young caregivers (most forum caregivers are sons and daughters of stroke survivors). With online comments provided directly from participants, the potential for ambiguity or distortion of patient views through transcription is reduced. This investigation suggests that there is potential for an online forum to add depth to understanding of stroke survivors' issues with medications. Themes identified from the online forum matched those which emerged from the qualitative interviews, suggesting that an online forum may well complement traditional data collection techniques for qualitative research.

Limitations of this research should also be acknowledged. While exploring barriers to medication adherence was the objective of the interview study, the online forum was not set up to investigate medication adherence and participants would not have focussed their conversations on barriers to adherence to secondary prevention medication. Younger forum participants were potentially more engaged with managing their condition than their offline counterparts, perhaps representing people with a good understanding of their health.

While interviewed participants contributed to one or more themes and were included in the interview study, several forum participants mentioning secondary prevention medications were excluded from the forum study because they did not provide enough details to allow the identification of a theme. Despite this, because of the wealth of information shared online and the high number of forum participants, the exclusion of several participants did not affect data collection within the forum study.

Comparisons with existing research

In agreement with our findings, investigations of online versus face to face focus group discussions concluded that both methods could be used to answer research questions, that online forum is more suited to communicate opinions and capturing participants' perspectives from a wide geographical area, [34] and, through anonymity, discussions of more personal issues. [44]

For peer review only

Issues around the potential reliability of forum data in answering the research question may also arise out of concerns about whether the data are skewed toward a specific participant group. As the use of the Internet to conduct behavioural research grows, the representativeness of participants' samples and issues will remain challenging, in spite of the advantages of increased accessibility to otherwise hidden populations.[20]

An online forum as source of data collection offers the opportunity for cross communication and shared support among participants. Through this forum, users can also draw on a personalised support system based on peer experiences and built trust. [45] At the same time, as shown in this study, online cross communication between participants can enhance understanding and add depth to the themes in qualitative research. With evidence that trust forms and develops on the online forum [46], there is the potential for this methodology to become an accepted and valued source of health information.[47]

Use of an online forum as a data collection technique in health care research raises potential ethical concerns around anonymity, privacy, confidentiality and informed consent compared to the more traditional qualitative approaches such as semi-structured interviews. [41 48]

Compared with face to face interviews where stroke survivors and caregivers were interviewed together, the forum offered an environment in which caregivers had the freedom to participate on their own (despite the existence of 'forums norms', i.e. codes of etiquette or accepted topics of conversations of forums[49]). In this context, participants may be more willing to express deeply held personal opinions and to discuss sensitive issues more freely, as described by Allen and colleagues.[5] The knowledge of such issues has the potential to inform and improve involvement of both patients and their caregivers in the decision making process, thus facilitating a collaborative approach around the use of medication, and encouraging effective medication taking behaviour.[50] An interesting observation was that interview survivors were more likely to follow GPs' instructions around medicines whereas forum survivors reported a shared approach to decisions. This identifies an interesting dynamic around how older and younger survivors view the practitioner role. Indeed previous research confirms that older patients look to the GP for support and view the practitioner as trustworthy and an ally in making healthcare decisions. [51]

For peer review only

Implications for research

The online forum represents a source of data collection suited to capturing the views of a younger stroke population who have access to online resources and to information from press outlets, which can potentially influence their attitudes to medications. The presence of younger patients and caregivers in online stroke forums offer insight for the development of interventions targeted to these groups. Indeed, research has shown that those who are younger with poorer mobility report most unmet needs, including in respect of medication taking.[52 53]

Stroke survivors who struggle with face to face communication but can communicate using technology such as a computer or mobile phone can provide insight on their needs, informing clinical interventions designed to improve medication taking in this patient group.

In agreement with the work on cancer forums, our work shows that the potential of online communities as a source of data is only beginning to be realised. Our findings suggest that data collected through an online forum complement traditional qualitative data collection techniques such as face to face interviews, giving researchers more confidence in using data from online forums. Online forum data also offered unprecedented ease of access to the caregiver perspective and the dynamic of their relationship with the stroke survivors in respect to barriers and facilitators to adherence to secondary prevention medications for stroke.

Conclusion

Both interviews and online forums are rich and useful sources of data and knowledge, revealing similar issues about patients' core experience. In uncovering additional themes the online forum may represent an important adjunct to traditional qualitative data collection methodologies.

Acknowledgements

We thank Jenni Burt and Chantal Balasooriya-Smeekens for their helpful comments on the manuscript.

For peer review only

Competing interests

None declared

Data sharing statement

No additional data are available

Author/s contribution

JJ contributed to the study design, data collection, data analysis and prepared the manuscript for submission. JM is a co-investigator on the study and commented on the manuscript. SS is a co-investigator on the study and commented on the manuscript. ADS contributed to the study design, data analysis and commented on the manuscript. All authors agreed on the final draft of the submitted manuscript.

For peer review only

References

1. nesta.org.uk. Peer Support: What is it and does it work? Summarising evidence from more than 1000 studies. : Nesta, 2015.

2. Why I turned to social media for help after a double lung transplant. Online health forums are increasingly used as a way of getting peer support and taking control of an illness. *The Guardian* 2016.

3. Pendry LF, Salvatore J. Individual and social benefits of online discussion forums. *Computers in Human Behavior* 2015;50:211-20. doi: http://dx.doi.org/10.1016/j.chb.2015.03.067

4. Ennis–O'Connor. How online patient communities are changing the face of cancer care. In: Blog B, ed., 2014.

5. Allen C VI, Kennedy A, Rogers A. Long-Term Condition Self-Management Support in Online Communities: A Meta-Synthesis of Qualitative Papers. *J Med Internet Res* 2016;18(3):e61. doi: 10.2196/jmir.5260

6. Im E-O, Chee W. Practical Guidelines for Qualitative Research Using Online Forums. *Computers, informatics, nursing : CIN* 2012;30(11):604-11. doi: 10.1097/NXN.0b013e318266cade

7. Im E-O, Chee W. An Online Forum As a Qualitative Research Method: Practical Issues. *Nursing research* 2006;55(4):267-73.

8. De Simoni A, Shanks A, Mant J, et al. Making sense of patients' internet forums: a systematic method using discourse analysis. *British Journal of General Practice* 2014;64(620):e178-e80. doi: 10.3399/bjgp14X677671

9. Balasooriya-Smeekens C, Bateman A, Mant J, et al. Barriers and facilitators to staying in work after stroke: insight from an online forum. *BMJ Open* 2016;6(4) doi: 10.1136/bmjopen-2015-009974

10. Office of National Statistics. Internet users in the UK: 2016 2016 [Available from: https://www.ons.gov.uk/businessindustryandtrade/itandinternetindustry/bulletins/internetusers/2016.

11. Ofcom. Internet use and Attitudes. Internet use and attitudes bulletin 2016, 2016.

For peer review only

12. Fox S. The Social Life of Health Information: Pew Research Centre; 2011 [Available from: http://www.pewinternet.org/2011/05/12/the-social-life-of-health-information-2011/.

13. Diaz JA, Griffith RA, Ng JJ, et al. Patients' Use of the Internet for Medical Information. *Journal of General Internal Medicine* 2002;17(3):180-85. doi: 10.1046/j.1525-1497.2002.10603.x

14. Ayers SL, Kronenfeld JJ. Chronic illness and health-seeking information on the Internet. *Health* 2007;11(3):327-47. doi: doi:10.1177/1363459307077547

15. McMullan M. Patients using the Internet to obtain health information: How this affects the patient–health professional relationship. *Patient Education and Counseling* 2006;63(1):24-28. doi: http://dx.doi.org/10.1016/j.pec.2005.10.006

16. Larner AJ. Searching the Internet for medical information: frequency over time and by age and gender in an outpatient population in the UK. *Journal of Telemedicine and Telecare* 2006;12(4):186-88. doi: doi:10.1258/135763306777488816

17. Andreassen HK, Bujnowska-Fedak MM, Chronaki CE, et al. European citizens' use of E-health services: A study of seven countries. *BMC Public Health* 2007 (Apr);10(7):53. doi: 10.1186/1471-2458-7-53

18. Taylor K. Connected health: How digital technology is transforming health and social care. *Deloitte Centre for Health Solutions* 2015

19. Moorhead SA, Hazlett DE, Harrison L, et al. A New Dimension of Health Care: Systematic Review of the Uses, Benefits, and Limitations of Social Media for Health Communication. *Journal of Medical Internet Research* 2013;15(4):e85. doi: 10.2196/jmir.1933

20. Rhodes SD, Bowie DA, Hergenrather KC. Collecting behavioural data using the world wide web: considerations for researchers. *Journal of Epidemiology and Community Health* 2003;57(1):68-73. doi: 10.1136/jech.57.1.68

21. De Simoni A, Shanks A, Balasooriya-Smeekens C, et al. Stroke survivors and their families receive information and support on an individual basis from an online forum: descriptive analysis of a population of 2348 patients and qualitative study of a sample of participants. *BMJ Open* 2016;6(4) doi: 10.1136/bmjopen-2015-010501

22. Kaufman S, Whitehead KA. Producing, ratifying, and resisting support in an online support forum. *Health (London)* 2016 (Feb) doi: 10.1177/1363459315628043

For peer review only

23. McKechnie V, Barker C, Stott J. The Effectiveness of an Internet Support Forum for Carers of People With Dementia: A Pre-Post Cohort Study. *J Med Internet Res* 2014;16(2):e68. doi: 10.2196/jmir.3166 [published Online First: 28.02.2014]

24. Malik S, Coulson NS. The Therapeutic Potential of the Internet: Exploring Self-Help Processes in an Internet Forum for Young People With Inflammatory Bowel Disease. *Gastroenterology Nursing* 2011;34(6):439-48. doi: 10.1097/SGA.0b013e318237a9ba

25. Alinia H, Moradi Tuchayi S, Farhangian ME, et al. Rosacea patients seeking advice: Qualitative analysis of patients' posts on a rosacea support forum. *Journal of Dermatological Treatment* 2016;27(2):99-102. doi: 10.3109/09546634.2015.1133881

26. Zhao J, Abrahamson K, Anderson JG, et al. Trust, empathy, social identity, and contribution of knowledge within patient online communities. *Behaviour & Information Technology* 2013;32(10):1041-48. doi: 10.1080/0144929X.2013.819529

27. Kenny AJ. Interaction in cyberspace: an online focus group. *Journal of Advanced Nursing* 2005;49(4):414-22. doi: 10.1111/j.1365-2648.2004.03305.x

28. Opdenakker R. Advantages and Disadvantages of Four Interview Techniques in Qualitative Research. *Forum: Qualitative Social Research* 2006;7(4)

29. Seymour WS. In the flesh or online? Exploring qualitative research methodologies. *Qualitative Research* 2001;1(2):147-68. doi: doi:10.1177/146879410100100203

30. Britten N. Qualitative research: Qualitative interviews in medical research. . *BMJ* 1995;311:251-53.

31. Souter C, Kinnear A, Kinnear M, et al. Optimisation of secondary prevention of stroke: a qualitative study of stroke patients' beliefs, concerns and difficulties with their medicines. *International Journal of Pharmacy Practice* 2014;22(6):424-32. doi: 10.1111/ijpp.12104

32. Seale C, Charteris-Black J, MacFarlane A, et al. Interviews and Internet Forums: A Comparison of Two Sources of Qualitative Data. *Qualitative Health Research* 2010;20(5):595-606. doi: doi:10.1177/1049732309354094

33. Synnot A, Hill S, Summers M, et al. Comparing Face-to-Face and Online Qualitative Research With People With Multiple Sclerosis. *Qualitative Health Research* 2014;24(3):431-38. doi: 10.1177/1049732314523840

For peer review only

34. Schneider SJ, Kerwin J, Frechtling J, et al. Characteristics of the Discussion in Online and Face-to-Face Focus Groups. *Social Science Computer Review* 2002;20(1):31-42. doi: 10.1177/089443930202000104

35. Eva María Murgado-Armenteros, Francisco José Torres-Ruiz and Manuela Vega-Zamora. Differences between Online and Face to Face Focus Groups, Viewed through Two Approaches *Journal of Theoretical and Applied Electronic Commerce Research* 2012;7(2):73-86.

36. Pawson R TN. Realistic Evaluation. London: Sage 1997.

37. Jamison J, Graffy J, Mullis R, et al. Barriers to medication adherence for the secondary prevention of stroke: a qualitative interview study in primary care. *British Journal of General Practice* 2016 doi: 10.3399/bjgp16X685609

38. Jamison J, Sutton S, Mant J, et al. A Barriers and facilitators to adherence to secondary stroke prevention medications after stroke: Analysis of survivors' and caregivers' views from an online stroke forum. *BMJOpen* 2017;7(7):e016814. . doi: 10.1136/bmjopen-2017-016814. [published Online First: July 16]

39. Horne R. Compliance adherence and concordance. In: Taylor K, Harding G, ed. Pharmacy Practice. London: Taylor & Franics 2001.

40. Britten N. Qualitative interviews in healthcare. In: Pope C MN, ed. Qualitative research in health care. 2nd ed. London: BMJ Books 1999:pp11-19.

41. Eysenbach G, Till JE. Ethical issues in qualitative research on internet communities. *BMJ* 2001;323(7321):1103-05. doi: 10.1136/bmj.323.7321.1103

42. Braun V, Clarke V. Using thematic analysis in psychology. *Qualitative Research in Psychology* 2006;3(2):77-101. doi: 10.1191/1478088706qp063oa

43. Horne R, Weinman J, Barber N, et al. Concordance, adherence and compliance in medicine – taking. In: R&D Report for the National Co-ordinating Centre for NHS Service Delivery and. Organisation R & D (NCCSDO) ed., 2005.

44. Zwaanswijk M, van Dulmen S. Advantages of asynchronous online focus groups and face-to-face focus groups as perceived by child, adolescent and adult participants: a survey study. *BMC Research Notes* 2014;7:756. doi: 10.1186/1756-0500-7-756

For peer review only

45. Brady E, Segar J, Sanders C. “You get to know the people and whether they’re talking sense or not”: Negotiating trust on health-related forums. *Social Science & Medicine (1982)* 2016;162:151-57. doi: 10.1016/j.socscimed.2016.06.029

46. Gill PS, Whisnant B. A qualitative assessment of an online support community for ovarian cancer patients. *Patient Related Outcome Measures* 2012;3:51-58. doi: 10.2147/PROM.S36034

47. Cole J, Watkins C, Kleine D. Health Advice from Internet Discussion Forums: How Bad Is Dangerous? *Journal of Medical Internet Research* 2016;18(1):e4. doi: 10.2196/jmir.5051

48. British Psychological Society. Ethics Guidelines for Internet-mediated research. Leicester, 2013.

49. Mudry TE, Strong T. Doing Recovery Online. *Qualitative Health Research* 2013;23(3):313-25. doi: 10.1177/1049732312468296

50. Belcher VN, Fried TR, Agostini JV, et al. Views of older adults on patient participation in medication-related decision making. *Journal of General Internal Medicine* 2006;21(4):298-303. doi: 10.1111/j.1525-1497.2006.00329.x

51. Butterworth JE, Campbell JL. Older patients and their GPs: shared decision making in enhancing trust. *The British Journal of General Practice* 2014;64(628):e709-e18. doi: 10.3399/bjgp14X682297

52. Evaluating the support stroke survivors get with daily medication taking in the community: interim results of a primary care survey. SAPC 2017; Warwick. EP1E.01.

53. Kersten P, Low JTS, Ashburn A, et al. The unmet needs of young people who have had a stroke: results of a national UK survey. *Disability and Rehabilitation* 2002;24(16):860-66. doi: 10.1080/09638280210142167

54. Bowker N, Tuffin K. Using the Online Medium for Discursive Research About People With Disabilities. *Social Science Computer Review* 2004;22(2):228-41. doi: doi:10.1177/0894439303262561

55. Mann C, & Stewart, F. Internet communication and qualitative research. Thousand Oaks, CA: Sage 2000.

56. Suler J. The Online Disinhibition Effect. *CyberPsychology & Behavior* 2004;7(3):321-26. doi: 10.1089/1094931041291295

For peer review only

57. DiCicco-Bloom B, Crabtree BF. The qualitative research interview. *Medical Education* 2006;40(4):314-21. doi: 10.1111/j.1365-2929.2006.02418.x

58. Stewart K, Williams M. Researching online populations: the use of online focus groups for social research. *Qualitative Research* 2005;5(4):395-416. doi: doi:10.1177/1468794105056916

59. Tanis M. Health-Related On-Line Forums: What's the Big Attraction? *Journal of Health Communication* 2008;13(7):698-714. doi: 10.1080/10810730802415316

60. White M, Dorman SM. Receiving social support online: implications for health education. *Health Education Research* 2001;16(6):693-707. doi: 10.1093/her/16.6.693

61. MacLean LM, Meyer M, Estable A. Improving Accuracy of Transcripts in Qualitative Research. *Qualitative Health Research* 2004;14(1):113-23. doi: doi:10.1177/1049732303259804

62. Wise K, Hamman B, Thorson K. Moderation, Response Rate, and Message Interactivity: Features of Online Communities and Their Effects on Intent to Participate. *Journal of Computer-Mediated Communication* 2006;12(1):24-41. doi: 10.1111/j.1083-6101.2006.00313.x

For peer review only

	Attribute	Online forum<br><br>*Recognised feature identified in the Literature (shaded cells)*<br>*Versus*<br>*Feature identified in the Present study*	Semi-structured interviews<br><br>*Recognised feature identified in the Literature (shaded cells)*<br>*Versus*<br>*Feature identified in the Present study*
**Context**	**Location**	Respondents from across a wide geographical area, can participate at own convenience.[54]	Interviewees geographically restricted.
		Patients' own home: UK	Patient's own home: East of England
	**Sampling**	Voluntary participation/ self-selection. Recruitment does not require collaboration between clinical sites or support of professional staff. [55]	Purposive recruitment in health care settings, guided by sampling techniques based on population demographics including age, gender and disability.
		Voluntary self-selection by participant: no sampling criteria, no restriction on age. Verification of stroke/TIA diagnosis not possible.<br>Most participants taking part within the first 5 years since the cerebrovascular event	Purposive sampling: GP screened, pre-defined criteria to achieve maximum spread of gender and disability.<br>Age: 55 years and over. Confirmed stroke/TIA diagnosis
**Mechanisms**	**Participation**		
		Multiple participants per conversation thread: stroke survivors or caregivers. Conversation possible between survivors, survivors and caregivers, or caregivers with other caregivers	Max 2 or 3 participants per single interview conversation: Researcher, stroke survivor and caregiver
	**Dynamic of interaction**	Discussion conducted remotely. Relative anonymity can encourage users to feel uninhibited.[5 56]<br>Likelihood of expressing honest opinions about sensitive issues.	Engagement can be actively encouraged.<br>Face to face approach enables development of rapport between interviewer and participant.[57]
		Less knowledge of participants, participants remain anonymous. No influence of researcher on participation.	Researcher's gain knowledge of interviewees, development of researcher-participant rapport, active encouragement of participation
	**Response contribution**	Longer conversations allow for a broader understanding of the subject matter and potential for greater depth.[58] Discussion threads generate reflection and greater description among users.[33]	Probing questions from researcher seeking clarification or to pursue a more detailed response.[40]
		Potential for significant individual contribution.<br>Responses shaped by other peer contributors.<br>More opportunity for self-reflection.<br>More frequent comments.	Maximum of two individual contributions (patients and caregiver).<br>Responses shaped by researcher interaction.<br>Less opportunity for self-reflection<br>In depth comments encouraged by

			researcher.
	**Timing of event**	Users can post repeatedly and frequently on many topics over a long period of time.	Interview is a single event occurring at one point in time Maximum of 2 contributors to the interview discussion.
		Single or multiple participation over time. Ability to contribute to discussion on multiple occasions/topics.	Single participatory event. Contribution fixed to a single time period.
	**Guidance**	User freedom to choose what to discuss, and how frequently to contribute to free-flowing discussion threads.[5] Posts created through peer to peer communication, without professionals' involvement and influence.[59] Response shaped by contribution of other survivors or caregivers.	Follows a pre-defined line of questioning. Several key questions define the area to be explored.[40] Researcher oversees the direction of conversation.
		Free or peer-guided discussions.	Guided conversation: responses to pre-defined questions in topic guide
	**Communication**	Permits broad accessibility and asynchronicity with online communication.[5, 60] Restricted to those with Internet access.[34]	Direct face to face, synchronous communication
		Indirect communication, via computer, no physical proximity, asynchronous	Direct communication, face to face, synchronous
**Outcomes**	**Activities**	No physical transcription is required; user contributions printed automatically, improving credibility of data.[7] Potential for inaccurate interpretation through misunderstanding nuances in the data may still exist.[7]	Transcription is key to representing the individual and dependability of data. Transcription opens data to misinterpretation or misunderstanding.[61] Rigour and accuracy in transcribing is integral to the analysis process, influencing the degree of dependability of data.[61]
		No audio recording. Automatic transcription printed directly from forum No field notes.	Interviews are audio recorded and interviews are transcribed. Potential for ambiguity through inaccurate transcribing Field notes taken during interviews
	**Reporting**	Forum posts are moderated before appearing online, effect on the data collected is relatively unknown. Moderation processes can influence engagement in online communities.[62]	
		3rd party moderation leading to possible exclusion of data	No exclusion of data prior to analysis

Footnote: Location: *Geographical area of the research.* Sampling: *Sampling method used to recruit participants.* Participation: *Individuals participating in conversations* Dynamic of interaction: *Knowledge of participant determined by level of engagement.* Response contribution: *Level of contribution to the conversation by individuals.* Timing of event: *Frequency of participation over time.* Guidance: *Level of conversation guidance and level of freedom to discuss* Communication: *Face to face versus distance communication*. Activities: *Need for audio recording and transcription activities*. Reporting: *Moderation of data before analysis*

For peer review only - http://bmjopen.bmj.com/site/about/guidelines.xhtml

Table 1. Key differences between an online forum approach and an interview approach to data collection

	Interviews	Online forum
**Sample characteristics**	**N [Median] (Range)**	**N [Median] (Range)**
Total participants	42	84
**Age**		
Survivor	[72] (61-93)	[50] (32-72)
*Caregiver	-	
**Gender**		
Male -Survivor	7	20
Female- Survivor	21	26
Not known - Survivor	-	3
*Caregiver of Male Survivor	4	20
*Caregiver of Female Survivor	10	12
Unknown gender and unknown identity	-	3
**Identity person posting**		
Stroke survivor	28	49
Caregiver	14	33
Not known	-	2
**Years since stroke**		
(0-12 mths)	4	37
(1-5 yrs)	14	25
(6-10 yrs)	6	4
(11-15 yrs)	2	2
(15+ yrs)	2	1
Unknown	-	15
**Type of stroke**		
TIA	14	-
Ischaemic stroke	13	-
Haemorrhagic stroke	1	-
**Caregiver identity**		
Daughter /son	2	20
Spouse	12	9
Other (/in law/ sister)	-	3
Unknown	-	1
**Number of posts about secondary prevention**		
		37 (1 participant)
		15 (1 participant)
		1 (44 participants)
		2 (19 participants)
		3 (6 participants)
Legend: * Refers to 'caregiver' in interviews and 'Patient talked about by caregiver' in the forum discussions		

Table 2. Characteristics of participants of the online forum and interview study

For peer review only

Perceptions				
**Key themes identified (interviews)**	**Interview quote^**	**Interview participant (Age, gender)**	**Forum quote***	**Forum participant♦ (Age, gender)**
	**Treatment necessity**		**Treatment necessity**	
**1. Knowledge of stroke and medications**	The importance of taking this exactly on time is trivial. I would probably survive for a week, if I didn't take the. For a month I'd probably survive. It would not make any difference in two days.<br><br>Whenever I've got a new pill or anything I'd read the instructions only because they've made a mistake before now, like for instance they gave me one which I''m allergic to… So I keep check of what I'm taking now.	Male, 86 yrs, N.03, ischaemic stroke<br><br>Male, 80 yrs N. 04, TIA	A female survivor commented that it was better to take a few extra tablets from the GP than to experience a stroke. Tablets were provided to prevent a further stroke, and she stressed that they shouldn't be stopped except on professional advice<br><br>A stroke survivor recalled being on 75mg of aspirin as well as beta blockers, however, his nephew who was a consultant surgeon, suggested that had he been taking warfarin instead of the aspirin he may not have suffered a second stroke<br><br>*(Bad press influences attitudes to medicines) A female survivor read about the hype around statins and stated she still didn't have confidence in them. She had read a research paper on statins suggesting they only added an extra 9 months of life. Her mum had been taking statins for 14 years and this still didn't prevent her arteries from clogging up*<br><br>*(Bad press influences attitudes to medicines) A survivor who had suffered 2 mini-strokes and been prescribed aspirin and cholesterol lowering statin but was refusing to take them because she had read in the press about bad side effects that they caused*	Female, age 51, age at stroke 51, N.17<br><br>Male, age 67, age at stroke 55, N.82<br><br>*Female, age 56, age at stroke 56, N.66*<br><br>Female, age unknown, age at stroke unknown, N.74
**2. Doubts about medicines**	I think aspirins are good for you. That's the one I fancy. Well it thins	Male, 75yrs, N.24, ischaemic	A survivor acknowledged statins were used to control cholesterol, but questioned whether high cholesterol	Male, age 67, age at stroke 55, n=70

For peer review only - http://bmjopen.bmj.com/site/about/guidelines.xhtml

	the blood and the blood it flows and that stops any clots so I do like to take it. I just don't see why I'm taking the other medication. I'm not fat or anything like that. I don't get very high blood pressure and well cholesterol, what is cholesterol..?	stroke	was actually a problem. He believed strokes occurred frequently, regardless of cholesterol levels. He talked about the 'Cholesterol Myth' having researched the topic on the Internet. He said he was feeling confused about the value of statins and taking these when in reality they weren't needed	
**3. Realisation of the importance of medicines**	At one time I wouldn't take a pill, I wouldn't even take an aspirin. Now I take it because I understand it keeps me alive. I just think it's fate, that's the way I look at it. If I stop taking medication I might as well lie down in the fast lane.	Male, 67yrs, N.12, ischaemic stroke	A male survivor already suffered 2 strokes and said it was impossible to ever fully recover from the experience. He said after his first stroke he was prescribed tablets he didn't take and he realizes this was a big mistake.	Male, age 67, age at stroke 55, n=82
Differing attitudes to medicines	Well I don't know what I'd be without taking them put it that way…because I've had a stroke and I've been fortunate. They keep me going, keep me on the straight and narrow.	Male, 73 yrs, N.11 ischaemic stroke	A female survivor felt it was better to take tablets from the GP than to experience another stroke. Tablets were provided to prevent another stroke and shouldn't be stopped except on professional advice	Female, age 51, age at stroke 51, N.17
	I refused it and I said well it's not because its rat poison. If you tell	Female, 71yrs, N.22	Another survivor remarked that, although being on pills was an inconvenience and she had stopped some	Female, age 52, age at stroke 52, N.76

For peer review only - http://bmjopen.bmj.com/site/about/guidelines.xhtml

	me I've got warfarin, I must be ill but if I take aspirin I can't be that ill.	TIA	medication, she continued to take aspirin and statin which she considered important	
	The only thing I like, I think aspirin's good for you, that's the only one I fancy. Well it thins the blood, and well thinning the blood makes it flow better and that stops any clots so I do like, like to take it	Male, 75 yrs N.25 ischaemic stroke	A survivor had suffered 2 strokes in the previous year, but none since commencing warfarin. She felt reassured by taking warfarin and worried about coming off the medication	Female, age 42, age at stroke 42, N.35
	I just take them because the hospital prescribed them. If the doctor prescribed them I probably wouldn't bother. I'd probably say forget about it. He's a consultant so he should know what he's talking about	Male, 67 yrs N.15 ischaemic stroke	A survivor described how he trusted his vascular surgeon who had changed his medication from warfarin to aspirin and statin. The survivor was happy to take aspirin and felt it would be good to continue as the surgeon also took it regularly, concluding it must be beneficial and would enable him to live longer.	Male, age 35, age at stroke 34, N.71
			A female survivor decided to reduce cholesterol using diet instead, because of side effects from medication. She felt that once the symptoms completely disappeared she wouldn't take a statin again. She said she would start taking olive oil and follow a healthy diet to keep her cholesterol balanced naturally. She said she would continue aspirin as it didn't seem to cause any side effects	Female, age 52, age at stroke 52, N.76
	**Treatment Concerns**		**Treatment Concerns**	
**4. How seriously people take medicines for secondary prevention of stroke**	I wouldn't take them because I still, to me, blood pressure and cholesterol tablets to me I don't see what they're doing for me	Male, 75 yrs, N.24, ischaemic stroke	A female survivor who had read bad reports about statins reported being nervous about them. She didn't want to jeopardise feeling good by taking medication that she wasn't convinced she needed	Female, age 54, age at stroke 54, N.37

For peer review only - http://bmjopen.bmj.com/site/about/guidelines.xhtml

	Well she gave me blood pressure pills and that but I didn't take them. I felt so, i didn't bother, pity really. But never mind. I do now. I'm religious about that. I'll have another stroke if I don't. Didn't want to put the family through that again.	Male, 65 yrs, N.02, ischaemic stroke	A survivor refused statins after her first stroke because of side effects. However, after suffering a second one she was now worried enough to take them.	Survivor, female, age 68, age at stroke 67, N.14
**5. Taking medications**<br>*Nonadherence to medicine*	Well now and again I forget the cholesterol because that's the one at night and it's the only one I take at night	Male, 67yrs, N.15 ischaemic stroke	A male survivor said he was on 2 tablets for blood pressure and that he continued to take one every day. But the other was a diuretic and having got fed up frequently running to the toilet, he decided to check his blood pressure every day and would skip the diuretic if it was fine.	Male, age unknown, age at stroke unknown, N.63
Trust in GP	So if the doctor says take ten pills a day, I'll, I'll do it….he makes the decision and erm he, he's the boss man as you might say, who knows what he's up to<br><br>I do exactly as the Dr tells me. He's the Dr isn't he. He should know better than what I do. I don't push them anymore and say well you know I don't like taking this	Male, 87yrs, N.8, TIA<br><br>Male, 80 yrs N.04, TIA	*A male survivor agreed with his doctor to stop taking a blood pressure tablet because of intolerable side effects, and his wife being a nurse made it easier. He felt strongly that doctors are there to advise not instruct.*<br><br>*(Collaborating with GP/ patient)*<br>*A caregiver said that her husband ceased taking medications except aspirin, because of side effects. He made this decision, together with the GP and stressed the importance of doing this before stopping tablets*	*Male, age unknown, age at stroke unknown, N.63*<br><br>Male, age 54, age at stroke 52, N.68

For peer review only - http://bmjopen.bmj.com/site/about/guidelines.xhtml

			Concern<br>*(Prescribing concerns)*<br>*A caregiver (sister) suggested that GPs shouldn't be paid for prescribing statins with the decision based on clinical judgement alone. The involvement of money could lead to medication being over prescribed for financial reasons*<br>*Concern*<br>*(Prescribing concerns)*<br>*A survivor described taking 9 pills a day for stroke and its side effects and felt that the GP should understand which were necessary. Following another appointment her consultant was furious about the medications she had been prescribed.*	Gender and age unknown, age at stroke unknown, N.78<br><br>Female, age 37, age at stroke 36
**Practicalities**				
	**Capability/ resources**		**Capability/ resources**	
**6. Ability to self-care**	My wife sorts it out and that's why I don't know so much about it you see she [taps].She puts them there, I take them and that's it	Male, 80 yrs, N.04, TIA	A caregiver stated that she was providing the stroke survivor with all of his medication due to his poor memory as a result of the stroke. She was now in complete control of his medication which she was happy about but it was difficult as he was a loved one and something she had no training for.<br><br>*(Caregiver as an advocate for the stroke survivor)*<br>*A female caregiver described consistently trying to have her husband's 40mg statin dosage reduced by his GP. As a result of the high dosage he was chronically tired, so he stopped taking statins*<br><br>*(Caregiver as an advocate for the stroke survivor)*<br>*A caregiver recommended being firm with GPs about being prescribed atorvastatin if simvastatin was not*	Female, Age 46, age at stroke 40, N.5<br><br>Male, age 54, age at stroke 52, N.68<br><br>Gender and age unknown, age at stroke unknown,

For peer review only

			tolerated, as atorvastatin was a bit more expensive but recommended by NICE guidelines as an alternative	N.18
**7. Taking medication**<br><br>*Problems swallowing*	The big ones, I, do actually feel I have to swallow two or three times to get them down	Male, 66yrs, N.10 TIA	A male survivor described 'swallow panic', i.e. fear of choking when trying to take Dipyridamol capsules. The user said it took around 3 months before he got over that.	Male, age unknown, age at stroke unknown, n=85
Accessing packaged medicines	Some of the, the pills are a hell of a trouble, you know the bubble wrap, flipping them out especially with my hands not as strong as they should be	Male, 87yrs, N.08, TIA	A caregiver (son in law) mentioned that despite the use of a nomad tray, tablets were still being taken from the wrong day with several days tablets being taken in a single day. The stroke survivor often didn't take the time to work out the days or to look at the calendar.	Male, age unknown, age at stroke unknown, n=111
	I'd have to rely on the wife to…cause I can't get them out the packet, just can't get your hands in	Male, 65yrs, N.02 Ischaemic stroke	A survivor agreed with another user about the problem with the size of dipyridamole tablets, which were getting stuck in the pill box organizer.	Female, age 46, age at stroke 45, N.30
**8. Medication routines**	I only remember to take the others if I take them out of the cupboard the night before and leave them on the top. If I didn't I would probably forget.. because it isn't the first thing that I think of	Male, 66yrs, N.10, TIA	A female survivor described keeping the pill box in a specific location in the house, such as by the kettle, which then acted as a reminder to check the medication box	Female, age 60, age at stroke 60,
	I usually take it around 5 o'clock which strangely enough is about the time that we feed the dog and normally speaking I take the medication then I get his dinner	Male, 68 yrs N.09 TIA	A survivor suggested using a white board and having method in place helped. She remembered taking her own medications through repetition or linking tablet use to another everyday activity	Female, age 54, age at stroke 46, N.19

For peer review only

For peer review only - http://bmjopen.bmj.com/site/about/guidelines.xhtml

**9. Changing medications**	They changed his medication to cheaper cholesterol and Dean was physically ill. He couldn't cope on it at all so he went back and the doctor said 'oh well it was just to try and they put him back on the others	Female, caregiver, N.24, age unknown	A survivor described being on 80 mg of simvastatin which they were happy with but that upon leaving hospital the dose was halved by the consultant which had very bad consequences, resulting in daily angina turns for a week. In the end he had to go back to his GP and be put back on the 80mg dose.	Female, age 53, age at stroke 50,
	She gave me an extra pill and I had a horrific night. She made an apology and said I'm sorry it took so long to get it right, but the fine tuning takes a bit of doing.	Male, 80 yrs N.04 TIA	A male survivor said he was taking up to 7 different blood pressure tablets and that it was unusual to only need a few tablets. He recommended going back to the GP as necessary to keep changing tablets until the correct combination was found.	Male, age 52, age at stroke 52, N.64
**10. Regimen complexity and burden of treatment**	I have to take 10 a day now altogether but I went up there (to the practice) to say can I get off some of these tablets, and I come back and I was on an extra one so I've not been up since	Male, 70yrs, N.13, TIA	*A caregiver (son) was asking advice on how to encourage medication taking. His mother was originally taking multiple tablets up to 4 times a day but that now she was refusing to take them all and he was upset by this. Persuading her to continue taking the most important tablets had taken hours to do.*	Female, age 77, age at stroke 77, N.9
	I've got yards of them. I don't know half the names I'm just told when to take them. That's one thing I'd like to do away with	Male, 73 yrs, N.11, Ischaemic stroke	*A caregiver (wife) described how her husband was adamant he was not prepared to take statins because he didn't have the time to keep going back to the GP for checkups. The caregiver said she was feeling helpless and wasn't sure what she could do about it*	Male, age 55, age at stroke 55, N.24
			(Burden of side effects on stroke survivor) A survivor described similar side effects from 3 different statins despite varying the medication dosage. She said tests confirmed this and she concluded long term use could result in problems that had a negative effect on her quality of life.	Female, age 34yrs, age at stroke 32, N.36

*Quote not transcribed - described to protect user confidentiality.

For peer review only - http://bmjopen.bmj.com/site/about/guidelines.xhtml

^Quote transcribed
♦ Demographic characteristics relate to the stroke survivors only (either talking in first person or talked about by a caregiver). Quotes in italics refer to additional themes identified in the online forum only

Table 3. Comparison of themes identified through analysis of semi-structured interviews and the online forum

For peer review only

For peer review only - http://bmjopen.bmj.com/site/about/guidelines.xhtml

Consolidated criteria for reporting qualitative studies (COREQ): 32-item checklist

**No**	**Item**	**Guide questions/description**	
**Domain 1: Research team and reflexivity**			
Personal Characteristics			
1.	Interviewer/facilitator	Which author/s conducted the interview or focus group?	N/A
2.	Credentials	What were the researcher's credentials? *E.g. PhD, MD*	BSc, MSc
3.	Occupation	What was their occupation at the time of the study?	Research Assistant/ PhD Student
4.	Gender	Was the researcher male or female?	Male
5.	Experience and training	What experience or training did the researcher have?	JJ is an experienced researcher with expertise in qualitative research.
Relationship with participants			
6.	Relationship established	Was a relationship established prior to study commencement?	N/A
7.	Participant knowledge of the interviewer	What did the participants know about the researcher? e.g. *personal goals, reasons for doing the research*	N/A
8.	Interviewer characteristics	What characteristics were reported about the interviewer/facilitator? e.g. *Bias, assumptions, reasons and interests in the research topic*	N/A
**Domain 2: study design**			
Theoretical framework			
9.	Methodological orientation and Theory	What methodological orientation was stated to underpin the study? *e.g. grounded theory, discourse analysis,*	Realist Evaluation Perspective. PAPA (Perception and Practicalities Approach. **Pg 7 + 8**

For peer review only

**No**	**Item**	**Guide questions/description**	
		ethnography, phenomenology, content analysis	
Participant selection			
10.	Sampling	How were participants selected? *e.g. purposive, convenience, consecutive, snowball*	N/A
11.	Method of approach	How were participants approached? e.*g. face-to-face, telephone, mail, email*	N/A
12.	Sample size	How many participants were in the study?	N/A
13.	Non-participation	How many people refused to participate or dropped out? Reasons?	N/A
Setting			
14.	Setting of data collection	Where was the data collected? e.*g. home, clinic, workplace*	Forum: online web site.<br>Interviews: in the patient/caregiver home. **Pg 6**
15.	Presence of non-participants	Was anyone else present besides the participants and researchers?	No
16.	Description of sample	What are the important characteristics of the sample? *e.g. demographic data, date*	Gender, age, stroke survivor/ caregiver, years since stroke occurred. **Table 2 Pg 25**
Data collection			
17.	Interview guide	Were questions, prompts, guides provided by the authors? Was it pilot tested?	N/A
18.	Repeat interviews	Were repeat interviews carried out? If yes, how many?	N/A
19.	Audio/visual recording	Did the research use audio or visual recording to collect the data?	N/A

For peer review only

**No**	**Item**	**Guide questions/description**	
20.	Field notes	Were field notes made during and/or after the interview or focus group?	N/A
21.	Duration	What was the duration of the interviews or focus group?	N/A
22.	Data saturation	Was data saturation discussed?	N/A
23.	Transcripts returned	Were transcripts returned to participants for comment and/or correction?	N/A
**Domain 3: analysis and findings**			
Data analysis			
24.	Number of data coders	How many data coders coded the data?	Interview: JJ-100%. **Pg. 6**
25.	Description of the coding tree	Did authors provide a description of the coding tree?	Table 3 reports themes identified and coded from the Interview/ Forum data. **Table 3. Pg 26-33**
26.	Derivation of themes	Were themes identified in advance or derived from the data?	Themes were generated from the data. **Pg. 6 and Pg. 7**
27.	Software	What software, if applicable, was used to manage the data?	Nvivo 10 **Pg. 7**
28.	Participant checking	Did participants provide feedback on the findings?	N/A
Reporting			
29.	Quotations presented	Were participant quotations presented to illustrate the themes / findings? Was each quotation identified? e.g. *participant number*	Forum: Yes, quotes presented as descriptions to protect confidentiality. Interview: Yes. Identification by unique participant number only. **Table 3 on Pg 26-33**

For peer review only

**No**	**Item**	**Guide questions/description**	
30.	Data and findings consistent	Was there consistency between the data presented and the findings?	Yes. **Table 3 Pg 26-33**
31.	Clarity of major themes	Were major themes clearly presented in the findings?	Yes. **Table 3 Pg 26-33**
32.	Clarity of minor themes	Is there a description of diverse cases or discussion of minor themes?	Yes. Key themes and sub-themes are reported. **Table 3 Pg 26-33**

For peer review only
